# Supplementary material for: A Secreted RNA Binding Protein Forms RNA-Stabilizing Granules in the Honeybee Royal Jelly
Source: Mol Cell. 2019 May 2;74(3):598–608.e6. doi: 10.1016/j.molcel.2019.03.010 (PMC6509358; doi:10.1016/j.molcel.2019.03.010)
Supplement: Document S1. Figures S1–S4 [file mmc1.pdf]

**Molecular Cell, Volume *74***

## Supplemental Information

# A Secreted RNA Binding Protein Forms RNA-Stabilizing Granules in the Honeybee Royal Jelly

**Eyal Maori, Isabela Cunha Navarro, Humberto Boncristiani, David J. Seilly, Konrad Ludwig Moritz Rudolph, Alexandra Sapetschnig, Chi-Chuan Lin, John Edward Ladbury, Jay Daniel Evans, Jonathan Luke Heeney, and Eric Alexander Miska**

# SUPPLEMENTAL INFORMATION

**Fig. S1 (related to Fig. 1) | Royal jelly proteins bind polymeric nucleic acids**

**(A)** Assessment of Alexa Fluor-488 labeled dsRNA (dsRNA*) stability in hive conditions to control for contaminating leakage of dsRNA solution into the newly secreted RJ. 50%, 25% and 10% sucrose solutions (w/v) were exposed to living bees for 30 min. Next, the sucrose solutions were mixed with dsRNA* (50 ng/ul final concentration) and incubated within hives for 0, 24, 48, 72 and 96 hours. dsRNA* integrity was analysed by gel electrophoresis of 10 $\mu$l samples. dsRNA* mixed in 10% sucrose solution could not be detected after 96 hours. Thus, dsRNA* was applied in 10% sucrose solution, and RJ was harvested 96 hours after the last dsRNA* application. **(B)** RT-PCR detection of dsRNA* in RJ samples harvested from control and dsRNA*-fed hives. **(C)** Worker jelly (WJ) proteins bind dsRNA. DsRNA-binding activity was tested by Electrophoretic Mobility Shift Assay (EMSA). Treatments included dsRNA mixed in RJ-buffer, 10% WJ mixed with dsRNA, 10% WJ was digested by PK and then mixed with dsRNA, 10% WJ was mixed with dsRNA and then digested by PK, 10% WJ was mixed with dsRNA and PK buffer, purified 27.3 µM BSA mixed with dsRNA, 10% WJ only, 10% WJ only digested by PK. 0.05 µM dsRNA was applied in all dsRNA-containing treatments. **(D)** The RJ dsRNA-binding proteins are soluble. dsRNA-binding activity was tested by EMSA. Treatments included dsRNA mixed in RJ buffer, 10% raw RJ mixed with dsRNA, 10% soluble RJ fraction mixed with dsRNA. 0.05 µM dsRNA was applied in all treatments. **(E)** Free nucleotides do not interfere with dsRNA-binding activity of RJ proteins. DsRNA-binding was tested in the presence of increasing concentrations of the negatively charged deoxynucleotides or nicotinamide adenine dinucleotide (NAD). 0.05 µM dsRNA was applied in all treatments. **(F)** The effect of RJ dilution factor on dsRNA band shift profile. DsRNA binding-activity was tested by EMSA. Constant dsRNA concentration was introduced to different RJ concentrations. Treatments included dsRNA mixed in RJ-buffer only and different raw and soluble RJ dilutions mixed with dsRNA. 0.05 µM dsRNA was applied in all treatments. **(G)** RJ proteins:dsRNA ratio affects dsRNA band shift profile. dsRNA-binding activity was tested by EMSA. Constant RJ concentration was introduced to increasing concentrations of dsRNA. Treatments included dsRNA mixed in RJ-buffer only and different

dsRNA concentrations mixed with 2% raw RJ (upper gel). In the gel below, gel electrophoresis of soluble RJ that was extracted from each sample post dsRNA mixture.

**Fig. S2 (related to Fig. 2) | MRJP-3 forms an oligomeric structure**

**(A)** MRJP-3 binds similarly 50 nt ssRNA and dsRNA carrying the same sequence. 42.8 μM proteins and 0.27 μM ssRNA or dsRNA were used in all RNA- and/or protein-containing treatments. **(B)** Purified MRJP-1 does not bind dsRNA as demonstrated by EMSA. dsRNA was incubated with decreasing concentrations of MRJP-3 and MRJP-1. Additional controls: MRJP-3 only, MRJP-1 only and dsRNA only. 0.08 μM dsRNA was applied in all dsRNA-containing treatments. **(C)** Graphical representation of MRJP-1 and MRJP-3. **(D)** Gel filtration analysis determines MRJP-3 molecular weight in RJ buffer. **(E)** Binding curve of Alexa Fluor-488 labeled MRJP-3 to MRJP-3 in RJ buffer. Calculated equilibrium disassociation constant (Kd) value is shown in dashed lines. **(F)** Evaluation of MRJP-3's concentration in RJ by comparative band intensity. RJ-1 and RJ-2: Two RJ samples collected from different hives. Equal sample volumes were loaded in all wells. Red and green spots represent treatments with similar band intensity. **(G)** The tandem-repeats region of MRJP-3 is required for RNA-binding. ssRNA-binding activity was tested by EMSA. Treatments included ssRNA only, full-length recombinant MRJP-3 (rMRJP-3) mixed with ssRNA, recombinant MRJP-3 lacking the repeats region (rMRJP3Δrepeats) mixed with ssRNA, rMRJP-3 mixed with ssRNA followed by proteinase K (PK) digestion, rMRJP-3 only. 0.3 μM ssRNA and 13.65 μM proteins were applied in all ssRNA- and/or protein-containing treatments.

**Fig. S3 (related to Fig. 3) | RNA mediates super-order assembly of MRJP-3 oligomers into large RNPs, and isolation of royal jelly RNA partners of MRJP-3**

**(A)** Super resolution OMX imaging of RNPs formed by MRJP-3 interaction with Alexa Fluor-488 labeled ssRNA or dsRNA. 0.2 μM ssRNA* or 0.03 μM dsRNA* were introduced to 15 μM unlabeled MRJP-3. Scale bar represents 1 μm. **(B)** RNA mediates super-order assembly of MRJP-3 oligomers, resulting in large RNPs formation. 0.15 μM ssRNA or ssRNA* were introduced to 42.8 μM unlabeled or Alexa Fluor-633 labeled MRJP-3 (MRJP-3 or MRJP-3* respectively). Scale bar represents 2 μm. **(C)** Bioanalyzer

Electropherograms of MRJP-3 bound RJ RNA. Complexes of RNA and biotinylated MRJP-3 were pulled down with strepatividine coated magnetic beads. Treatments also included RNA pull-down with biotinylated BSA or with beads only. **(D)** VDV-1 coverage plot. The y-axis shows the per-base read coverage for each RNA-seq library across the genome of VDV-1 (green: MRJP-3 bound RNA; blue: total RJ RNA). Positive and negative coverage values represent viral RNA that corresponds to the plus (sense) or minus (antisense) VDV-1 genome, respectively. **(E)** MRJP-3 binds putative long tRNA-dsRNA fragments (≥25 bp).

**Fig. S4 (related to Fig. 4) | MRJP-3 bound dsRNA is protected from digestion of RNaseA, but not RNaseIII**

**(A)** MRJP-3 bound dsRNA is protected from RNaseA digestion. Treatments included dsRNA mixed with MRJP-3, dsRNA mixed with MRJP-3 followed by incubation with RNaseA, dsRNA mixed with MRJP-1, dsRNA mixed with MRJP-1 followed by incubation with RNaseA, dsRNA mixed in RJ buffer and dsRNA mixed in RJ buffer followed by incubation with RNaseA. 0.04 µM dsRNA and 42.8 µM MRJP-3 or MRJP-1 were used in all dsRNA- and protein-containing treatments. RNase challenge was performed by introducing 0.2 ug RNaseA followed by 3 hours incubation at room temperature. **(B)** MRJP-3 bound dsRNA is digested by RNase-III. Treatments included dsRNA mixed with MRJP-3, dsRNA mixed with MRJP-3 followed by incubation with RNase-III, dsRNA mixed with MRJP-1, dsRNA mixed with MRJP-1 followed by incubation with RNase-III, dsRNA mixed in RJ buffer and dsRNA mixed in RJ buffer followed by incubation with RNase-III. 0.04 µM dsRNA and 42.8 µM MRJP-3 or MRJP-1 were used in all dsRNA- and protein-containing treatments. RNase-III challenge was performed by introducing $2x10^{-2}$ units RNase-III followed by 3 hours incubation at room temperature. **(C)** MRJP-3 RNPs are susceptible to RNase-III. Three images of RNPs formed with dsRNA* with or without RNase-III. 0.04 µM dsRNA* and 42.8 µM MRJP-3 were used in all dsRNA*- and protein-containing treatments. RNase challenge was performed by introducing $2x10^{-2}$ units RNase-III followed by 3 hours incubation in room temperature. Scale bar represents 20 µm. **(D)** Labelled dsRNA stability in animals soaking assay conditions. Gel electrophoresis of animals soaking solution treated with PK.

Fig. S1

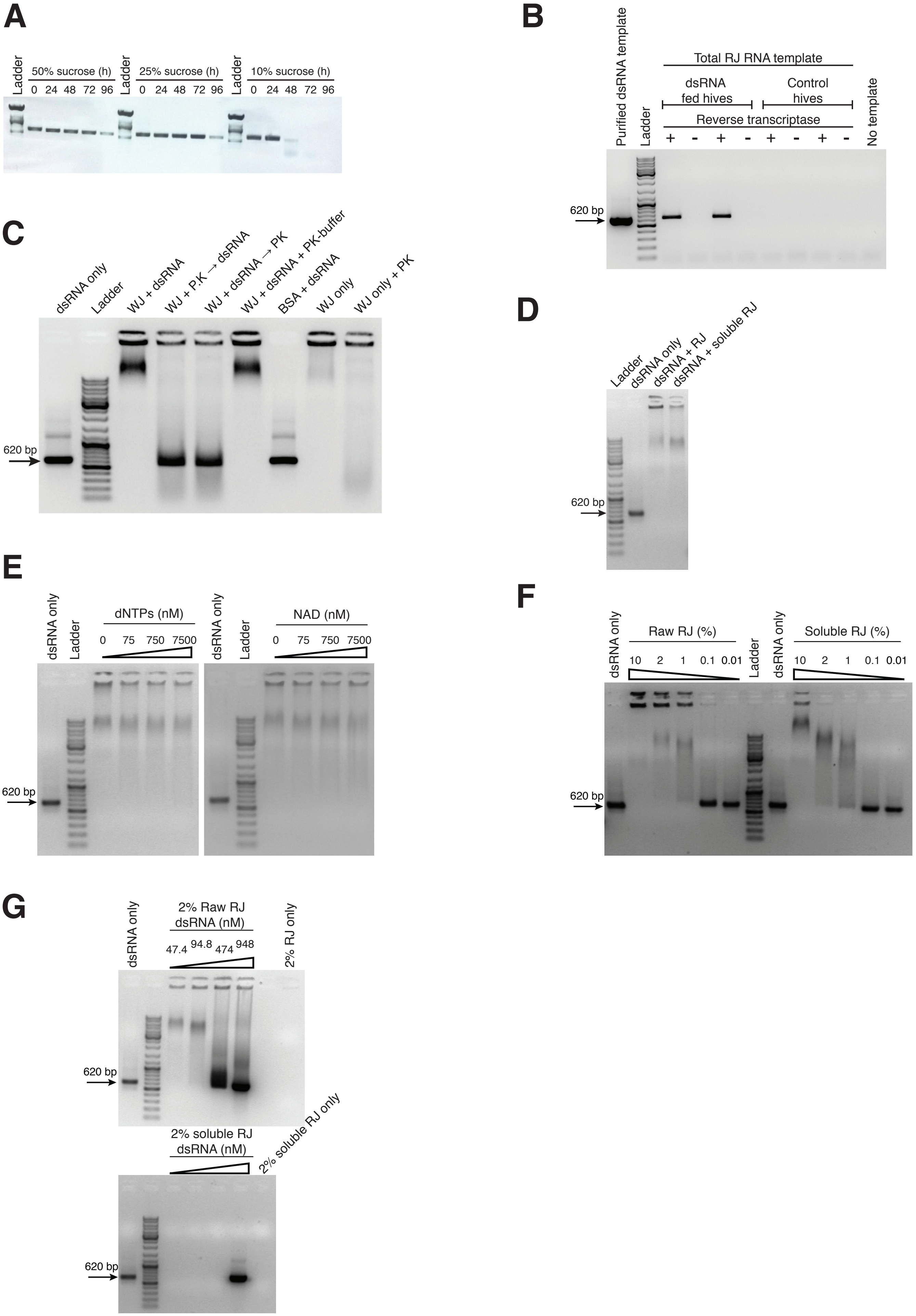
A
Ladder
50% sucrose (h)
0 24 48 72 96
25% sucrose (h)
10% sucrose (h)
B
Purified dsRNA template
Total RJ RNA template
dsRNA fed hives
Control hives
Reverse transcriptase
No template
620 bp
C
dsRNA only
WJ + dsRNA
WJ + P.K → dsRNA
WJ + dsRNA → PK
WJ + dsRNA + PK-buffer
BSA + dsRNA
WJ only
WJ only + PK
D
dsRNA + RJ
dsRNA + soluble RJ
E
dNTPs (nM)
0 75 750 7500
NAD (nM)
F
Raw RJ (%)
10 2 1 0.1 0.01
Soluble RJ (%)
G
2% Raw RJ
dsRNA (nM)
47.4 94.8 474 948
2% RJ only
2% soluble RJ
2% soluble RJ only

Fig. S2

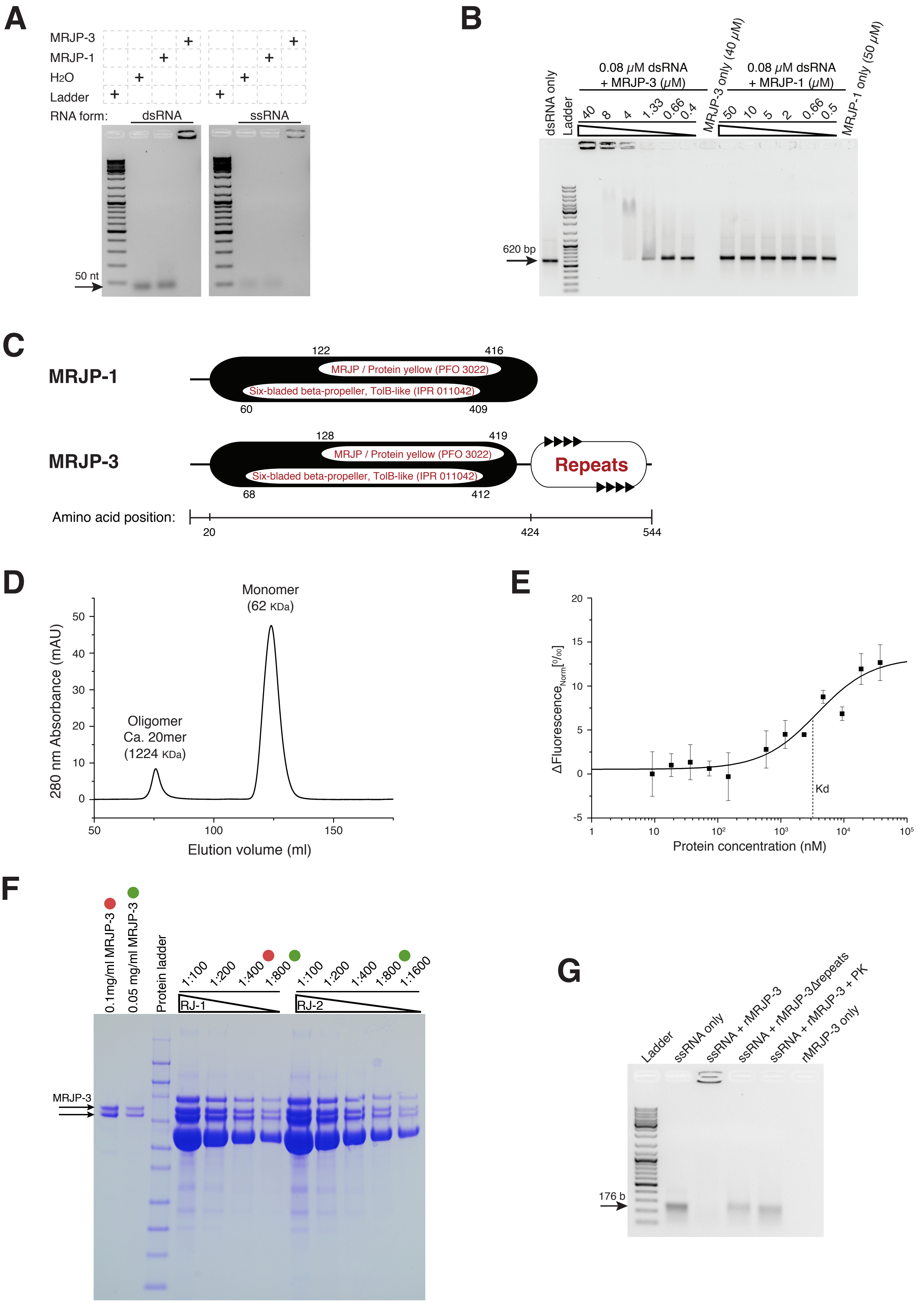

A
MRJP-3
MRJP-1
H2O
Ladder
RNA form:
dsRNA
ssRNA
50 nt
B
dsRNA only
Ladder
0.08 μM dsRNA + MRJP-3 (μM)
40 8 4 1.33 0.66 0.4
MRJP-3 only (40 μM)
0.08 μM dsRNA + MRJP-1 (μM)
50 10 5 2 0.66 0.5
MRJP-1 only (50 μM)
620 bp
C
MRJP-1
122
416
MRJP / Protein yellow (PFO 3022)
Six-bladed beta-propeller, TolB-like (IPR 011042)
60
409
MRJP-3
128
419
MRJP / Protein yellow (PFO 3022)
Six-bladed beta-propeller, TolB-like (IPR 011042)
68
412
Repeats
Amino acid position:
20
424
544
D
Monomer (62 KDa)
Oligomer Ca. 20mer (1224 KDa)
280 nm Absorbance (mAU)
Elution volume (ml)
E
ΔFluorescence Norm [‰]
Kd
Protein concentration (nM)
F
0.1mg/ml MRJP-3
0.05 mg/ml MRJP-3
Protein ladder
1:100 1:200 1:400 1:800
RJ-1
1:100 1:200 1:400 1:800 1:1600
RJ-2
MRJP-3
G
Ladder
ssRNA only
ssRNA + rMRJP-3
ssRNA + rMRJP-3Δrepeats
ssRNA + rMRJP-3 + PK
rMRJP-3 only
176 b

Fig. S3

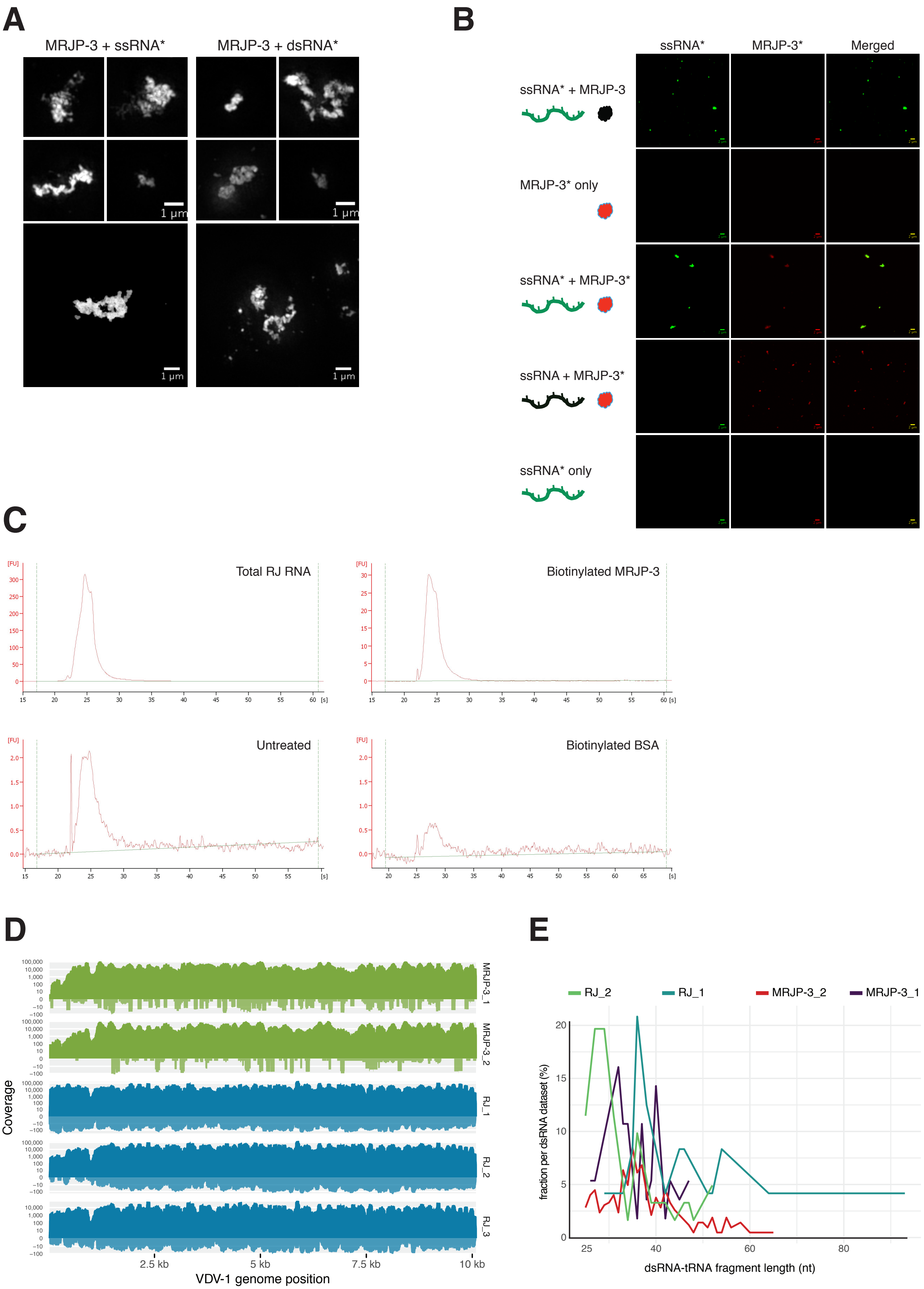
A
MRJP-3 + ssRNA*
MRJP-3 + dsRNA*
1 µm
1 µm
1 µm
1 µm
B
ssRNA*
MRJP-3*
Merged
ssRNA* + MRJP-3
MRJP-3* only
ssRNA* + MRJP-3*
ssRNA + MRJP-3*
ssRNA* only
C
Total RJ RNA
Biotinylated MRJP-3
Untreated
Biotinylated BSA
[FU]
[s]
D
Coverage
MRJP-3_1
MRJP-3_2
RJ_1
RJ_2
RJ_3
2.5 kb
5 kb
7.5 kb
10 kb
VDV-1 genome position
E
RJ_2
RJ_1
MRJP-3_2
MRJP-3_1
fraction per dsRNA dataset (%)
dsRNA-tRNA fragment length (nt)

Fig. S4

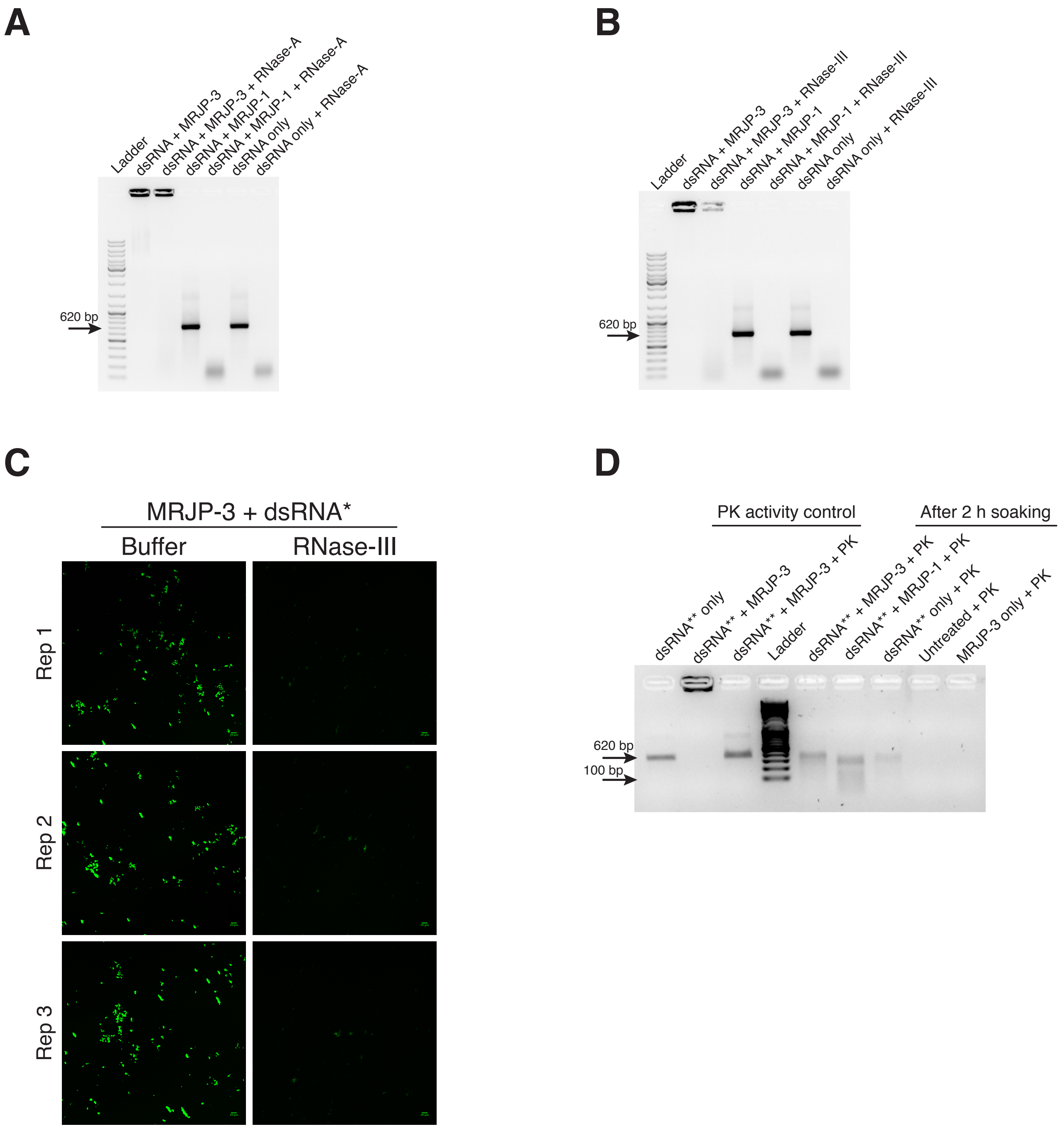
A
Ladder
dsRNA + MRJP-3
dsRNA + MRJP-3 + RNase-A
dsRNA + MRJP-1
dsRNA + MRJP-1 + RNase-A
dsRNA only
dsRNA only + RNase-A
620 bp
B
Ladder
dsRNA + MRJP-3
dsRNA + MRJP-3 + RNase-III
dsRNA + MRJP-1
dsRNA + MRJP-1 + RNase-III
dsRNA only
dsRNA only + RNase-III
620 bp
C
MRJP-3 + dsRNA*
Buffer
RNase-III
Rep 1
Rep 2
Rep 3
D
PK activity control
After 2 h soaking
dsRNA** only
dsRNA** + MRJP-3
dsRNA** + MRJP-3 + PK
Ladder
dsRNA** + MRJP-3 + PK
dsRNA** + MRJP-1 + PK
dsRNA** only + PK
Untreated + PK
MRJP-3 only + PK
620 bp
100 bp
