# Supplementary material for: A Secreted RNA Binding Protein Forms RNA-Stabilizing Granules in the Honeybee Royal Jelly
Source: Mol Cell. 2019 May 2;74(3):598–608.e6. doi: 10.1016/j.molcel.2019.03.010 (PMC6509358; doi:10.1016/j.molcel.2019.03.010)
Supplement: Table S4. List of Nucleic Acids, Related to STAR Methods — Information of nucleic acids used in this study. [file mmc5.pdf]

Supplementary Table 2A: Information of nucleic acid sequences used in this study

No	Sequence name	RNA/ DNA form	Length (nt)	Synthesis method	Corresponding NCBI accession no.	Source
1	dsRNA-Fluc	dsRNA	617	*In vitro* transcription	AB261988	In house
2	ssRNA-CTCT	ssRNA	176	*In vitro* transcription	N/A	In house
3	10 nt ss-oligo	ssRNA	10	Chemical synthesis	N/A	Integrated DNA Technologies (IDT)
4	15 nt ss-oligo	ssRNA	15	Chemical synthesis	N/A	Integrated DNA Technologies (IDT)
5	18 nt ss-oligo	ssRNA	18	Chemical synthesis	N/A	Integrated DNA Technologies (IDT)
6	20 nt ds-oligo	dsRNA	20	Chemical synthesis	N/A	Integrated DNA Technologies (IDT)
7	21 nt ss-oligo	ssRNA	21	Chemical synthesis	N/A	Integrated DNA Technologies (IDT)
8	22 nt ss-oligo	ssRNA	22	Chemical synthesis	N/A	Integrated DNA Technologies (IDT)
9	23 nt ss-oligo	ssRNA	23	Chemical synthesis	N/A	Integrated DNA Technologies (IDT)
10	50 nt ss-oligo	ssRNA	50	Chemical synthesis	N/A	Integrated DNA Technologies (IDT)
11	50 nt ds-oligo	dsRNA	50	Chemical synthesis	N/A	Integrated DNA Technologies (IDT)
12	dsRNA-*unc-22*	dsRNA	1365	*In vitro* transcription	NM_001268674	In house

dsRNA-Fluc DNA template sequence (excluding the T7 promoters; 5' → 3')

AAAACGCTTCCATCTTCCAGGGATACGACAAGGATATGGGCTCACTGAGACTACATCAGCTATTCTGATTACAC
CCGAGGGGGATGATAAACCGGGCGCGGTCGGTAAAGTTGTTCCATTTTTTGAAGCGAAGGTTGTGGATCTGGAT
ACCGGGAAAACGCTGGGCGTTAATCAGAGAGGCGAATTATGTGTCAGAGGACCTATGATTATGTCCGGTTATGT
AAACAATCCGGAAGCGACCAACGCCTTGATTGACAAGGATGGATGGCTACATTCTGGAGACTTAGCTTACTGGG
ACGAAGACGAACACTTCTTCATAGTTGGCCGCTTGAAGTCTTTAATTAAATACAAAGGATATCAGGTGGCCCCC
GCTGAATTGGAATCGATATTGTTACAACACCCCAACATCTTCGACGCGGGCGTGGCAGGTCTTCCCGACGATGA
CGCCGGTGAACTTCCCGCCGCCGTTGTTGTTTTGGAGCACGGAAAGACGATGACGGAAAAAGAGATCGTGGATT
ACGTCGCCAGTCAAGTAACAACCGCGAAAAAGTTGCGCGGAGGAGTTGTGTTTGTGGA

ssRNA-CTCT DNA template sequence (excluding the T7 promoters; 5' → 3')

CTCTCTCTCTCTCTCTCTCTCTCTCTCTCTCTCTCTCTCTCTCTCTCTCTCTCTCTCTCTCTCTCTCTCTCTCT
CTCTCTCTCTCTCTCTCTCTCTCTCTCTCTCTCTCTCTCTCTCTCTCTCTCTCTCTCTCTCTCTCTCTCTCTCT
CTCTCTCTCTCTCTCTCTCTCTCTCTCT

10 nt ss-oligo sequence (5' → 3')

ACUGUUACUA

15 nt ss-oligo sequence (5' → 3')

GGCUCACUGUUACUA

18 nt ss-oligo sequence (5' → 3')
UGUGUUUAUAGCGGAUUU

20 nt ss-oligo sequence (5' → 3')
UGUGUUUAUAGCGGAUUUUG

21 nt ss-oligo sequence (5' → 3')
UGUGUUUAUAGCGGAUUUUGA

22 nt ss- and ds-oligo sequence (5' → 3')
TCATATGGGCTCACTGTTACTA

23 nt ss-oligo sequence (5' → 3')
AGGUAAGAUGUUGGUAUAGCUGA

50nt ss- and ds-oligo sequence (5' → 3')
ACGCUUCUAACUUCAAGAUUUUACACAAUCAUAUGGGCUCACUGUUACUA

dsRNA-*unc-22* DNA template sequence (excluding the T7 promoters; 5' → 3')
AGACCGGCAGATCTGATATCATCGATGAATTCGAGCTCCACCGCGGTGGCGGCCGCTCTAGAACTAGTGGATCC
ACCGGTTCCATGGCTAGCCACGTGACGCGTGGATCCCCCGGGCTGCAGGAATTCGATTACCAACGGTTTTGGAA
GGGACAAACATTCCGTTGAGGTCATTGTGCTTGGAAAACCATTCTAGCCCCATTGGGACCTTTGGAAGTGTCGA
ATGTCTACGAAGATCGCGCAGATTTGGAGTGGAAAGTACCAGAAGATGACGGAGGTGCTCCAATTGATCATTAT
GAAATCGAAAAGATGGATTTGGCAACTGGAAGATGGGTCCCATGTGGAAGAAGTGAAACAACAAAGACCACAGT
TCCAAATCTTCAACCCGGGCACGAATACAAATTCCGTGTCAGAGCTGTGAACAAGGAGGGAGAATCCGATCCAC
TCGCAACCAACACCGCAATCCTCGCCAAGAACCCATACGAGGTTCCAGGAAAAGTTGACAAGCCGGAATTGGTG
GACTGGGATAAGGATCATGTTGATCTTGCATGGAATGCTCCAGACGATGGTGGTGCACCAATTGAAGCTTTCGT
CATTGAGAAGAAGGATAAGAATGGACGATGGGAAGAAGCTCTCGTTGTTCCAGGAGATCAGAAAACAGCAACTG
TTCCAAATCTTAAGGAGGGAGAAGAATATCAATTCAGAATTTCTGCTCGTAACAAGGCTGGAACTGGAGATCCT
TCTGATCCTTCTGATCGTGTTGTTGCGAAGCCAAGAAACCTTGCTCCAAGAATTCATCGTGAAGATCTTTCTGA
TACAACTGTCAAGGTCGGAGCCACTCTCAAGTTCATTGTTCATATTGATGGTGAGCCAGCACCAGATGTAACAT
GGTCATTCAATGGAAAAGGAATCGGAGAGAGCAAGGCTCAAATTGAAAATGAGCCATACATCTCGAGATTTGCT
TTGCCAAAGGCACTTCGTAAGCAAAGTGGAAAATATACCATCACTGCAACCAACATTAATGGAACTGACAGTGT
CACTATCAATATCAAGGTAAAAAGCAAGCCAACGAAACCAAAGGGACCAATCGAGGTAACTGATGTCTTCGAAG
ATCGTGCAACTCTTGACTGGAAACCACCAGAGGATGACGGAGGAGAGCCAATTGAGTTCTATGAAATTGAAAAG
ATGAACACCAAGGACGGAATCTGGGTTCCATGTGGACGTAGTGGAGATACCCACTTCACAGTCGATTCACTCAA
CAAGGGAGATCATTAATCAAGCTTATCGATACCGTCGACCTCGAGGGGGGGCCCGGTACCCAATTCG

Supplementary Table 2B: List of primers in this study

**Primers & purpose (5' → 3')**	**Amplified sequence**	**Product size (bp)**
DsRNA-Fluc DNA template synthesis & RT-PCR detection:<br><br>Forward:<br>CTAATACGACTCACTATAGGGCGAaaaacgcttccatcttcca<br>Reverse:<br>CTAATACGACTCACTATAGGGCGAtccacaaacacaactcctc	AB261988	617
DsRNA-*unc-22* DNA template synthesis:<br><br>Forward:<br>CTAATACGACTCACTATAGGGagaccggca<br>Reverse:<br>CTAATACGACTCACTATAGGGcgaattgggt	Unc-22 RNAi clone ZK617.1 (Source Bioscience)	1365

Comment: T7 RNA-pol promoter sequence is highlighted in capital letters
