# Supplementary material for: A Secreted RNA Binding Protein Forms RNA-Stabilizing Granules in the Honeybee Royal Jelly
Source: Mol Cell. 2019 May 2;74(3):598–608.e6. doi: 10.1016/j.molcel.2019.03.010 (PMC6509358; doi:10.1016/j.molcel.2019.03.010)
Supplement: Document S2. Article plus Supplemental Information [file mmc6.pdf]

Short Article

Molecular Cell

A Secreted RNA Binding Protein Forms RNA-Stabilizing Granules in the Honeybee Royal Jelly

Graphical Abstract

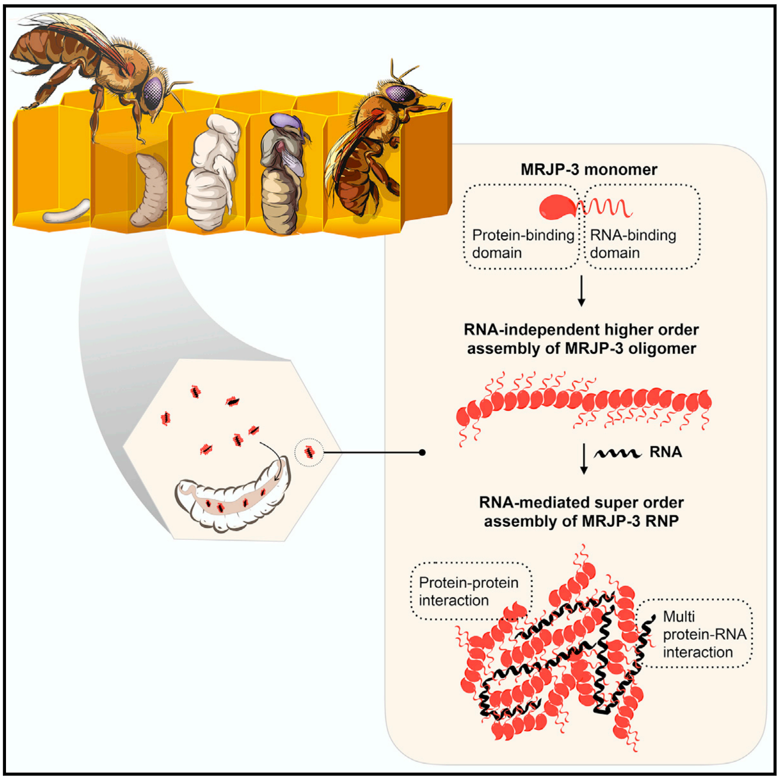

Highlights

- Worker and royal jellies harbor robust RNA-binding activity
- Major royal jelly protein 3 (MRJP-3) is the dietary RNA-binding factor
- RNA stimulates higher-order assembly of MRJP-3 into extracellular RNP granules
- MRJP-3 granules concentrate, stabilize, and enhance environmental RNA bioavailability

Authors

Eyal Maori, Isabela Cunha Navarro, Humberto Boncristiani, ..., Jay Daniel Evans, Jonathan Luke Heeney, Eric Alexander Miska

Correspondence

eyalmm@gmail.com (E.M.), jlh66@cam.ac.uk (J.L.H.), eam29@cam.ac.uk (E.A.M.)

In Brief

Honeybees share RNA between individuals through secretion and ingestion of worker and royal jellies. Maori et al. identify a jelly factor, MRJP-3, that forms extracellular ribonucleoprotein granules that concentrate, protect, and enhance RNA uptake. They propose that MRJP-3 facilitates RNA transfer that drives social immunity and signaling among bees.

Maori et al., 2019, Molecular Cell *74*, 598–608
May 2, 2019 Crown Copyright © 2019 Published by Elsevier Inc.
https://doi.org/10.1016/j.molcel.2019.03.010

Molecular Cell
Short Article

A Secreted RNA Binding Protein Forms RNA-Stabilizing Granules in the Honeybee Royal Jelly

Eyal Maori,[1,2,*] Isabela Cunha Navarro,[1,2] Humberto Boncristiani,[3] David J. Seilly,[4] Konrad Ludwig Moritz Rudolph,[1,2,7] Alexandra Sapetschnig,[1,2] Chi-Chuan Lin,[5] John Edward Ladbury,[5] Jay Daniel Evans,[6] Jonathan Luke Heeney,[4,*] and Eric Alexander Miska[1,2,7,8,*]
[1]The Gurdon Institute, University of Cambridge, Cambridge, CB2 1QN, UK
[2]Department of Genetics, University of Cambridge, Cambridge CB2 3EH, UK
[3]Department of Entomology and Nematology, University of Florida, Gainesville, FL 32611, USA
[4]Department of Veterinary Medicine, University of Cambridge, Cambridge CB3 0ES, UK
[5]School of Molecular and Cellular Biology, University of Leeds, Leeds LS2 9JT, UK
[6]USDA-ARS Bee Research Lab, BARC-E Building, 306 Center Road, Beltsville, MD 20705, USA
[7]Wellcome Sanger Institute, Wellcome Genome Campus, Cambridge CB10 1SA, UK
[8]Lead Contact
*Correspondence: eyalmm@gmail.com (E.M.), jlh66@cam.ac.uk (J.L.H.), eam29@cam.ac.uk (E.A.M.)
https://doi.org/10.1016/j.molcel.2019.03.010

SUMMARY

RNA flow between organisms has been documented within and among different kingdoms of life. Recently, we demonstrated horizontal RNA transfer between honeybees involving secretion and ingestion of worker and royal jellies. However, how the jelly facilitates transfer of RNA is still unknown. Here, we show that worker and royal jellies harbor robust RNA-binding activity. We report that a highly abundant jelly component, major royal jelly protein 3 (MRJP-3), acts as an extracellular non-sequence-specific RNA-aggregating factor. Multivalent RNA binding stimulates higher-order assembly of MRJP-3 into extracellular ribonucleoprotein granules that protect RNA from degradation and enhance RNA bioavailability. These findings reveal that honeybees have evolved a secreted dietary RNA-binding factor to concentrate, stabilize, and share RNA among individuals. Our work identifies high-order ribonucleoprotein assemblies with functions outside cells and organisms.

INTRODUCTION

Distinct biochemical activities are often found in specialized subcellular compartments. These compartments are demarcated either by a membrane barrier or through the process of phase transition, which drives macromolecular condensation and formation of membrane-less organelles (Hyman et al., 2014). Multivalent protein-RNA interactions induce and maintain the assembly of such membrane-less ribonucleoprotein (RNP) organelles (Lin et al., 2015; Maharana et al., 2018). RNP assemblies (or condensates) occur in both the nucleus and the cytoplasm. Nuclear condensates include Cajal bodies, nuclear speckles, and the nucleoli, and cytoplasmic examples are stress granules, processing bodies (P bodies), and P granules. To date, with the exception of plasma lipoproteins, high-order RNP assemblies have been detected only inside cells.

Protein-coding and non-coding RNA can spread among cells and tissues of an organism. Such mobile RNA has been documented in plants and animals (Molnar et al., 2010; Winston et al., 2002). Furthermore, RNA transfer among organisms has been reported among fungi, plants, and animals (Buck et al., 2014; Cai et al., 2018; Shahid et al., 2018; Zhu et al., 2017). Transmissible RNA has been associated mainly with RNAi to modulate gene expression and immune responses in the recipient organisms. However, much about the biology and mechanisms of mobile and transmissible RNA remains unknown.

The honeybee (*Apis mellifera*) plays a key role in agriculture, pollinating a large number of crops that feed humans and farm animals. In recent years, elevated honeybee losses have become a major global concern. Bee colony losses have been linked to various biotic stressors, including the mite Varroa destructor, Israeli acute paralysis virus (IAPV), and other viruses (Maori et al., 2007; McMenamin and Genersch, 2015). Previously, we reported on RNAi-based ingestion systems for the control of IAPV and the Varroa mite (Garbian et al., 2012; Maori et al., 2009). Field trials of the double-stranded RNA (dsRNA)-IAPV treatment showed a potential prolonged disease resistance in treated hives, lasting several months after the final dsRNA application, at a time when the treated bees would have been replaced by new generations (Hunter et al., 2010). Following this observation, we revealed that bees are able to share RNA among individuals as well as generations, through secretion and ingestion of worker and royal jelly (RJ) (Maori et al., 2019). On the basis of the presence of naturally occurring RNA populations in the jellies and the transmission of biologically active RNA between honeybees, we hypothesized that the jelly has evolved means to facilitate environmental transfer of RNA.

 Crown Copyright © 2019 Published by Elsevier Inc.
This is an open access article under the CC BY-NC-ND license (http://creativecommons.org/licenses/by-nc-nd/4.0/).

RJ is a larval food source, secreted by the hypopharyngeal and mandibular glands of young workers. Whereas worker larvae are fed on RJ only for the first 3 days of development, queens are nourished on RJ their entire lives. Therefore, this secretion plays a central role in honeybee caste differentiation. RJ is an acidic (pH 3.5–4.5) aqueous solution of proteins, sugars, lipids, vitamins, salts, free amino acids, and RNA (Maori et al., 2019; Stocker et al., 2005). Major royal jelly proteins (MRJPs) represent about 90% of the total RJ protein content. Nine MRJPs are encoded by the *Apis mellifera* genome (MRJP-1 to MRJP-9), but their physiological functions are not well understood. MRJP-3 is a polymorphic protein that represents 10%–15% of the total RJ protein content (Furusawa et al., 2008). The polymorphic MRJP-3 alleles vary in the number of repeat units within the tandem-repeat region (TRR) at the C terminus, and it is speculated that the basic TRR has been selected for an increase in nitrogen storage to enhance nutrition (Albertová et al., 2005). MRJP-3 expression is upregulated following bacterial infection (Scharlaken et al., 2008), and purified protein modulates mice immune responses *in vitro* and *in vivo* (Okamoto et al., 2003).

Here we show that MRJP-3, an abundant jelly ingredient, is a secreted non-sequence-specific RNA-binding protein and that multivalent RNA binding mediates the transition of MRJP-3 into extracellular RNP (eRNP) granules that concentrate, stabilize, and enhance environmental RNA uptake.

RESULTS

RJ Proteins Bind RNA

To investigate the role of RJ in RNA transmission, we fed beehives either on sucrose only or on sucrose mixed with labeled dsRNA (dsRNA*). On day 5, queens were removed to initiate queen rearing and RJ secretion (Figure 1A). To exclude any possible contamination of the newly secreted RJ with the dsRNA* in the sucrose solution, we harvested RJ 4 days after the last dsRNA feed (Figure S1A). We then confirmed that RJ from dsRNA-fed hives contains full-length dsRNA* using RT-PCR (Figure S1B). Next, we asked how the dsRNA might be distributed in the RJ. We visualized the dsRNA* in RJ using immunohistochemistry and observed that the RNA was not distributed homogeneously in the jelly but rather concentrated in ~0.5–10 μm granule structures (Figure 1B). We then asked if the non-dispersive RNA localization is due to association with a jelly factor. To test this, we performed electrophoretic mobility shift assays (EMSAs) and found that RJ proteins bind dsRNA (Figure 1C). We observed a similar dsRNA-binding activity also in worker jelly (WJ) (Figure S1C). However, as the collection of WJ is challenging and because all bee larvae are fed on RJ for the first 3 days of life (Wright et al., 2018), we focused on RJ for all subsequent experiments.

We detected RNA-binding activity in both raw and soluble RJ extracts (see STAR Methods; Figure S1D). Binding appeared to be specific to polymeric nucleic acids, as the negatively charged deoxynucleotides (dNTPs) or nicotinamide adenine dinucleotides (NADs) were not able to compete with dsRNA binding (Figure S1E). Next, we tested dsRNA binding in serial dilutions of raw and soluble RJ extracts. RJ showed detectable levels of RNA binding down to 1% dilution (Figure S1F). RJ concentration affected complex size (Figure S1F), suggesting a multivalent mode of RNA binding by jelly proteins. We noticed that the addition of RNA induces precipitation in RJ extracts. To validate the effect of RNA on RJ, we introduced increasing amounts of dsRNA to raw 2% RJ extracts. Interestingly, titrating dsRNA into raw RJ extracts triggered precipitation, which mostly dissolved back into solution at high dsRNA concentration (Figure 1D). Consistently, EMSA demonstrated that increasing RNA concentration results in decreased RNP size (Figure S1G). We conclude that multivalent RNA-binding jelly protein(s) form RNPs with the ability to aggregate; the protein/RNA ratio affects both RNP size and solubility, somewhat analogous to the phenomena of phase transition and polyclonal antibody-antigen precipitation dynamics (Heidelberger and Kendall, 1935; Hyman et al., 2014).

MRJP-3 Is the RNA-Binding Jelly Protein

To identify RNA-binding proteins in RJ, we fractionated the jelly using fast protein liquid chromatography (FPLC) and screened the unbound and eluted fractions for dsRNA-binding activity by EMSA (see STAR Methods). By using cation exchange chromatography (68 screened fractions) followed by hydroxyapatite chromatography (82 screened fractions), we isolated a single protein with RNA-binding activity: MRJP-3. Because we also observed RNA-binding activity in WJ (Figure S1C), we tested and confirmed that MRJP-3 is indeed present in both jellies, using liquid chromatography followed by mass spectrometry (LC-MS/MS). Twenty-eight unique peptides (overall 538 peptides) covering 70% of MRJP-3 were detected in RJ, and 27 unique peptides (overall 250 peptides) covering 63.3% of the protein were detected in WJ (Table S1). MRJP-3 consists of three domains: an N-terminal secretion signal, an MRJP/protein-yellow domain, and a TRR (Figure 1E). A taxonomic tree for MRJP-3 suggests that although the MRJP/protein-yellow domain is widely conserved, the TRR emerged in the *Apis* genus and is associated with jelly-secreting bees only (Figure 2A).

Purified MRJP-3 bound to both dsRNA and single-stranded RNA (ssRNA) (Figure 2B; Figure S2A). As observed in the jelly extracts, increasing quantity of RNA results in a gradual decrease in the size of the MRJP-3 RNP complex, again indicating multivalent MRJP-3:RNA binding (Figure 2B). Major royal jelly protein 1 (MRJP-1), which shares 80% amino acid sequence similarity with MRJP-3, assembles into an oligomeric form (Tamura et al., 2009) but lacks both the TRR and RNA-binding activity (Figures S2B and S2C; Figure 2B). Using gel filtration, we found that in the absence of RNA, purified MRJP-3 also assembles into a higher-order oligomeric form, composed of ~20 monomer units (Figure S2D). We used microscale thermophoresis (MST) to confirm MRJP-3 self-assembly and showed that a defined oligomer size was obtained (Figure S2E). We introduced increasing concentrations of non-labeled MRJP-3 to fluorescently labeled monomer and observed elevation of the signal as the labeled protein bound to the concentration-dependent higher-order MRJP-3 oligomers. The signal stabilized at once the concentration of added MRJP-3 reached approximately 40 μM, confirming that the labeled MRJP-3 could not bind further to increasing concentrations of added protein (i.e., a stable oligomer was present). The MST analysis also showed that MRJP-3 self-association has

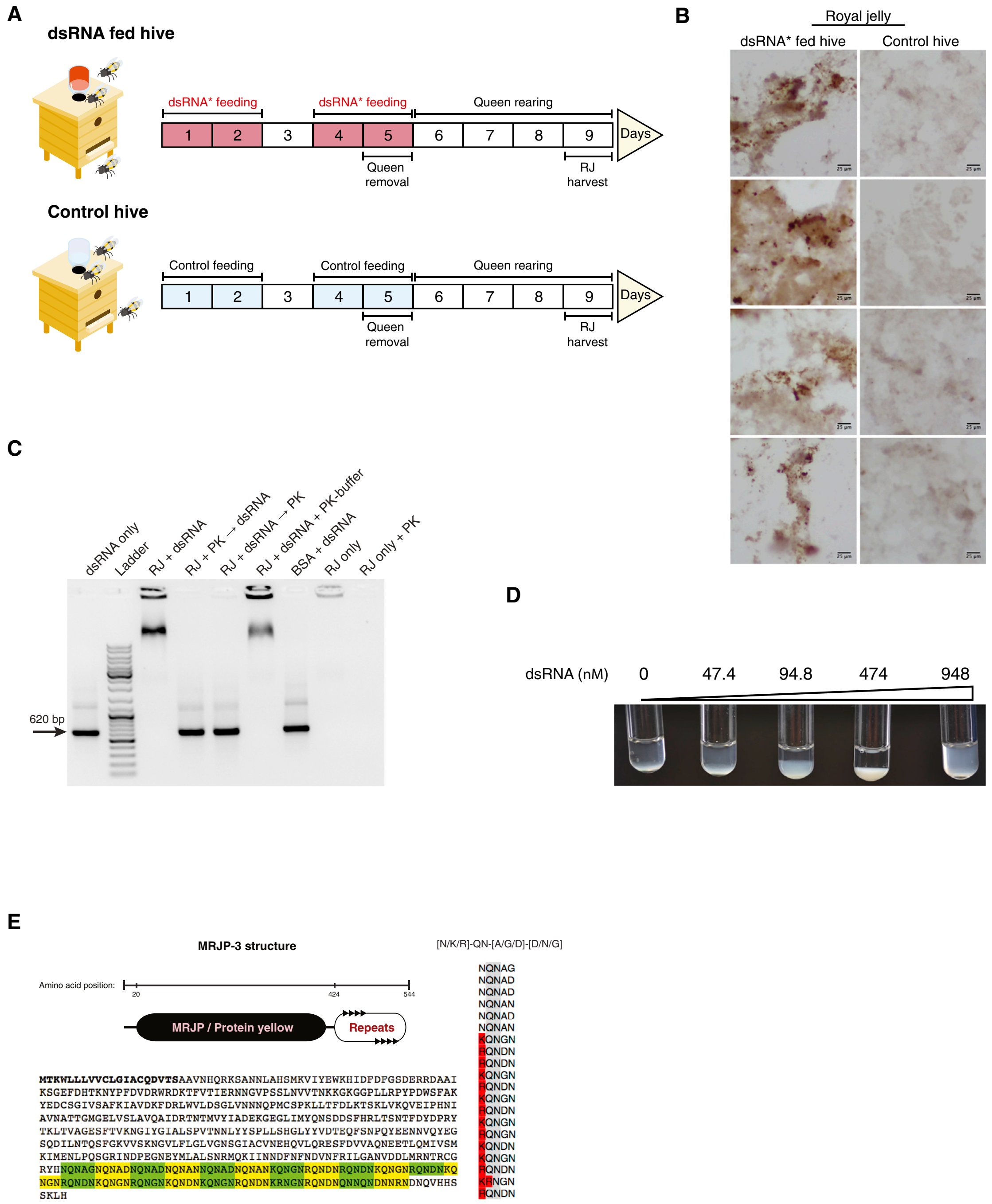

Figure 1. The Honeybee Jelly Harbors RNA-Binding Activity

(A) Experimental design for RNA detection in RJ. Hives were fed with a 10% sucrose solution with or without the addition of Alexa Fluor-488-labeled dsRNA (dsRNA*).

(B) Immunohistochemistry-based detection of dsRNA* in RJ samples, which were reacted with Alexa Fluor-488 antibody. Scale bar represents 25 μm.

(C) RJ proteins bind dsRNA. dsRNA-binding activity was tested using EMSA. Treatments included dsRNA mixed in RJ buffer, 10% RJ mixed with dsRNA, 10% RJ digested by Proteinase K (PK) and then mixed with dsRNA, 10% RJ mixed with dsRNA and then digested by PK, 10% RJ mixed with dsRNA and PK buffer, 27.3 μM purified BSA mixed with dsRNA, 10% RJ only, and 10% RJ only digested by PK. dsRNA (0.05 μM) was applied in all dsRNA-containing treatments.

(D) Precipitation dynamics of dsRNA-protein complexes in RJ. Two percent RJ was mixed with increasing dsRNA concentrations.

(E) MRJP-3 and its prion-like TRR. Amino acid sequence in bold: secretion signal peptide. Amino acid sequence highlighted in color: tandem repeats. Alignment of the tandem repeats, QN (in gray) and positively charged amino acids (in red).

See also Figure S1 and Table S1.

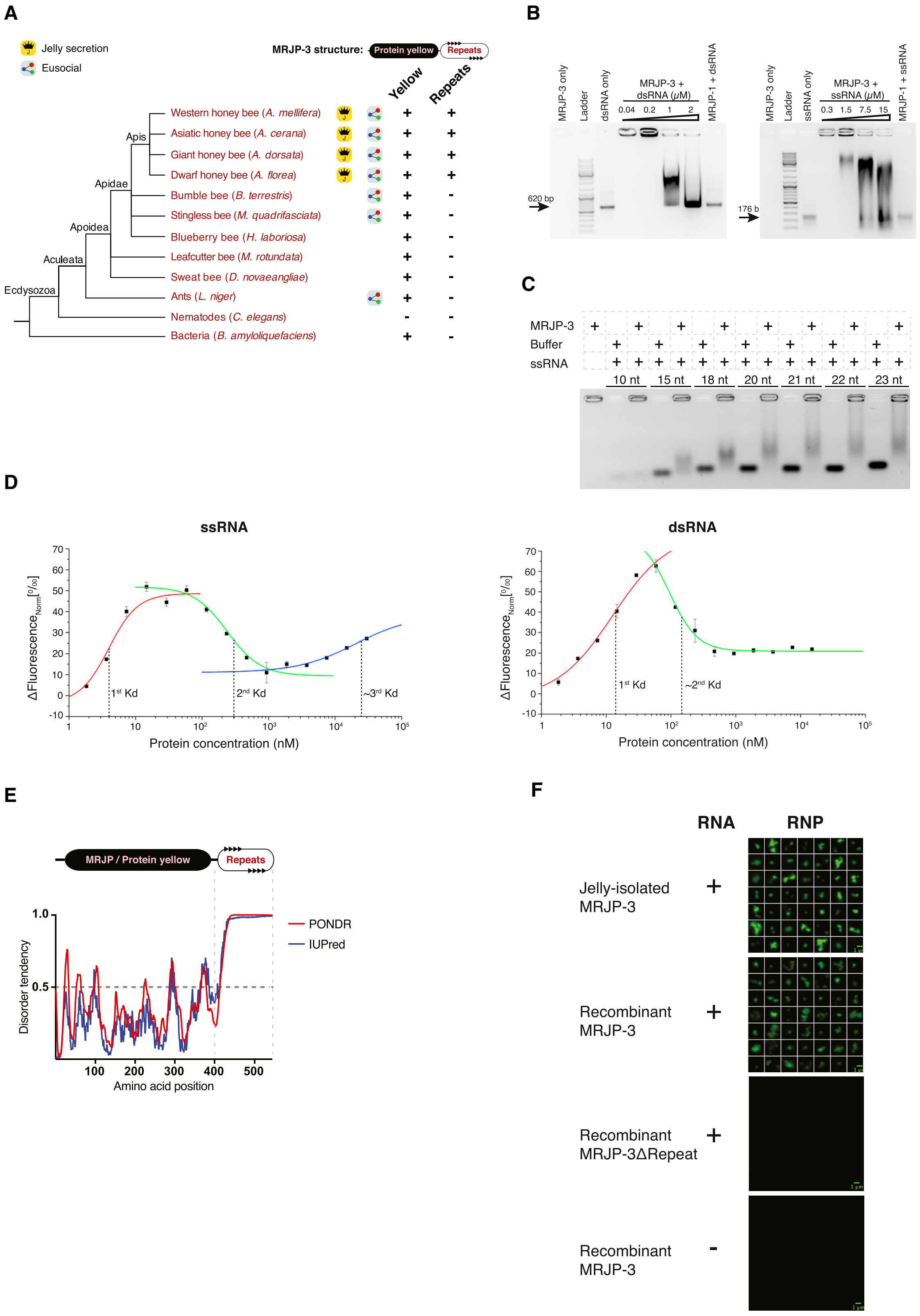

(legend on next page)

an apparent K_d of 3.5 μM (Figure S2E). Finally, we estimated the MRJP-3 concentration in RJ to be ~40 mg/mL (648 μM) (Figure S2F). We therefore conclude that MRJP-3 is a highly abundant RNA-binding oligomer in native RJ.

MRJP-3 binds ssRNA and dsRNA of different length and sequence, indicating a non-sequence-specific mode of binding (Figure 2B; Figure S2A). However, we tested whether nucleic acid length could be a limiting factor for binding and found that a minimal length of 18 nt was required for efficient MRJP-3 RNA binding (Figure 2C). We used MST to characterize the binding of MRJP-3 to ssRNA and dsRNA and found that introducing MRJP-3 to both RNA types results in multi-phasic binding curves (Figure 2D). Three discrete binding events were observed for ssRNA. By fitting these individual binding events, with the assumption that they were independent of one another, we obtained K_d values of 4 nM, 300 nM, and 25 μM for ssRNA. We observed two distinct binding events for dsRNA and identified K_d values of 11 nM and 150 nM. The tight interactions at low protein concentrations suggest that the MRJP-3 monomer has high affinity to both unstructured and duplexed RNA. The defined K_d values also indicate that MRJP-3 binds ssRNA and dsRNA in different self-associated states: before, during, and after the completion of oligomerization. Because the K_d for self-association was 3.5 μM and the MRJP-3 concentration in RJ is ~26 times more than the highest K_d value measured with RNA, RNA is expected to be bound to the fully self-associated MRJP-3 oligomers in native conditions.

The repetitive unit of MRJP-3's TRR includes poly glutamine-asparagine amino acids (Figure 1E), which are characteristic of intrinsic disordered proteins and prion-like domains (Halfmann et al., 2011; Michelitsch and Weissman, 2000). Intrinsic protein disorder analysis supports this feature of the TRR region (Figure 2E). The TRR is enriched also with positively charged amino acids such that whereas the full-length protein has a pI of 6.47, the TRR (UniProt: Q17060; aa 424–523) has a pI of 10.10, implying that this region is positively charged in the acidic RJ environment (pH 3.5–4.5). To test if the positively charged TRR plays a role in RNA binding, we produced recombinant MRJP-3 that lacks the TRR and demonstrated, using EMSA, that the prion-like TRR is required for the RNA-binding activity (Figure S2G).

MRJP-3 and RNA Form Dynamic RNP Granules

Both jelly-purified and recombinant full-length MRJP-3, when mixed with RNA, formed a high–molecular weight RNP complex (Figure 2B; Figures S2A and S2G). To gain further insight into these RNP complexes, we imaged jelly-purified or full-length recombinant MRJP-3 mixed with labeled RNA and observed the formation of 0.1–4 μm RNP granules. The prion-like TRR, required for RNA binding, is also required for the formation of these RNP granules (Figure 2F). Both ssRNA and dsRNA mediated super-order assembly of the oligomeric MRJP-3 into large RNPs (Figure S3A). To determine whether the MRJP-3 RNP granules are dynamic, we first incubated MRJP-3 with labeled RNA and then introduced increasing quantities of non-labeled competitor RNA. We observed a gradual decrease in labeled RNP size, indicating that RNA is reversibly bound by MRJP-3 or that high RNA concentrations de-assemble the RNP granules by affecting the multivalent protein-RNA interactions (Figure 3A).

EMSA revealed that different RNA/MRJP-3 ratios result in different binding patterns (Figures 2B and 3A). To test directly whether the RNA/MRJP-3 ratio affects MRJP-3 RNP size, we mixed increasing quantities of labeled RNA with fixed MRJP-3 concentrations and imaged the resulting RNPs. Increasing the RNA/MRJP-3 ratio initially increased the RNP granules size until a point was reached after which the complexes started to decrease in size (Figure 3B), thus supporting a multivalent mode of RNA binding. To provide further evidence that RNA mediates super-order assembly of MRJP-3 oligomers into RNP granules, we used two colors to differentially label MRJP-3 (MRJP-3*; red) and RNA (ssRNA*; green) (Figure S3B). ssRNA* alone was homogeneously dispersed in buffer. However, green loci appeared when ssRNA* was mixed with MRJP-3, demonstrating RNA condensation and RNP formation. When ssRNA* was mixed with MRJP-3*, green-red RNPs were formed and co-localized, demonstrating that RNA and MRJP-3 physically interact, and the presence of RNA leads to super-order assembly of MRJP-3 oligomers into RNP condensates. We then tested whether RNA triggers the formation of MRJP-3 RNP granules in RJ-like conditions (Figure 3C). When ssRNA* is mixed with soluble RJ fractions containing MRJP-3*, red foci were formed and co-localized with the green RNA signal. Introducing non-labeled ssRNA resulted in the formation of red foci, demonstrating that the RNA itself, not the labeling fluor, mediated super-order assembly of MRJP-3 oligomers.

MRJP-3 Binds Naturally Occurring Jelly RNA

To determine whether MRJP-3 binds naturally occurring RJ RNA, we incubated biotinylated MRJP-3 or biotinylated BSA in RJ, to pull down any associated RNA. MRJP-3 bound RNAs had similar bioanalyzer electropherogram profiles to total RJ

Figure 2. MRJP-3 Is a Multivalent RNA-Binding Oligomer

(A) Taxonomy tree analysis suggests that the MRJP-3 tandem-repeats region evolved in the genus *Apis* and is associated with jelly secretion.

(B) Purified MRJP-3 binds dsRNA and ssRNA as demonstrated by EMSA. MRJP-3 was incubated with increasing concentrations of dsRNA or ssRNA. Additional controls: dsRNA and ssRNA only, MRJP-1 mixed with 43.1 nM dsRNA or 0.3 μM ssRNA, and MRJP-3 only. Protein (42.8 μM) was applied in all MRJP-3- or MRJP-1-containing treatments.

(C) MRJP-3 efficiently binds ssRNA that is 18 nt and longer. Binding activity was tested using ssRNA substrates with different lengths and analyzed by EMSA. ssRNA (19.4 pmol) and proteins (42.8 μM) were applied in all ssRNA- and/or protein-containing treatments.

(D) Binding curves of Alexa Fluor-488-labeled 22 nt ssRNA and dsRNA to MRJP-3 in RJ buffer conditions (left and right curves, respectively). Calculated and estimated equilibrium K_d values are shown as dashed lines.

(E) The TRR of MRJP-3 is predicted to be intrinsically disordered by the PONDR VSL2 and IUPred algorithms.

(F) The TRR of MRJP-3 is required for RNP formation. Proteins (13.6 μM) and ssRNA* (0.2 μM) were applied in all RNA- and/or protein-containing treatments. Scale bar represents 1 μm.

See also Figure S2.

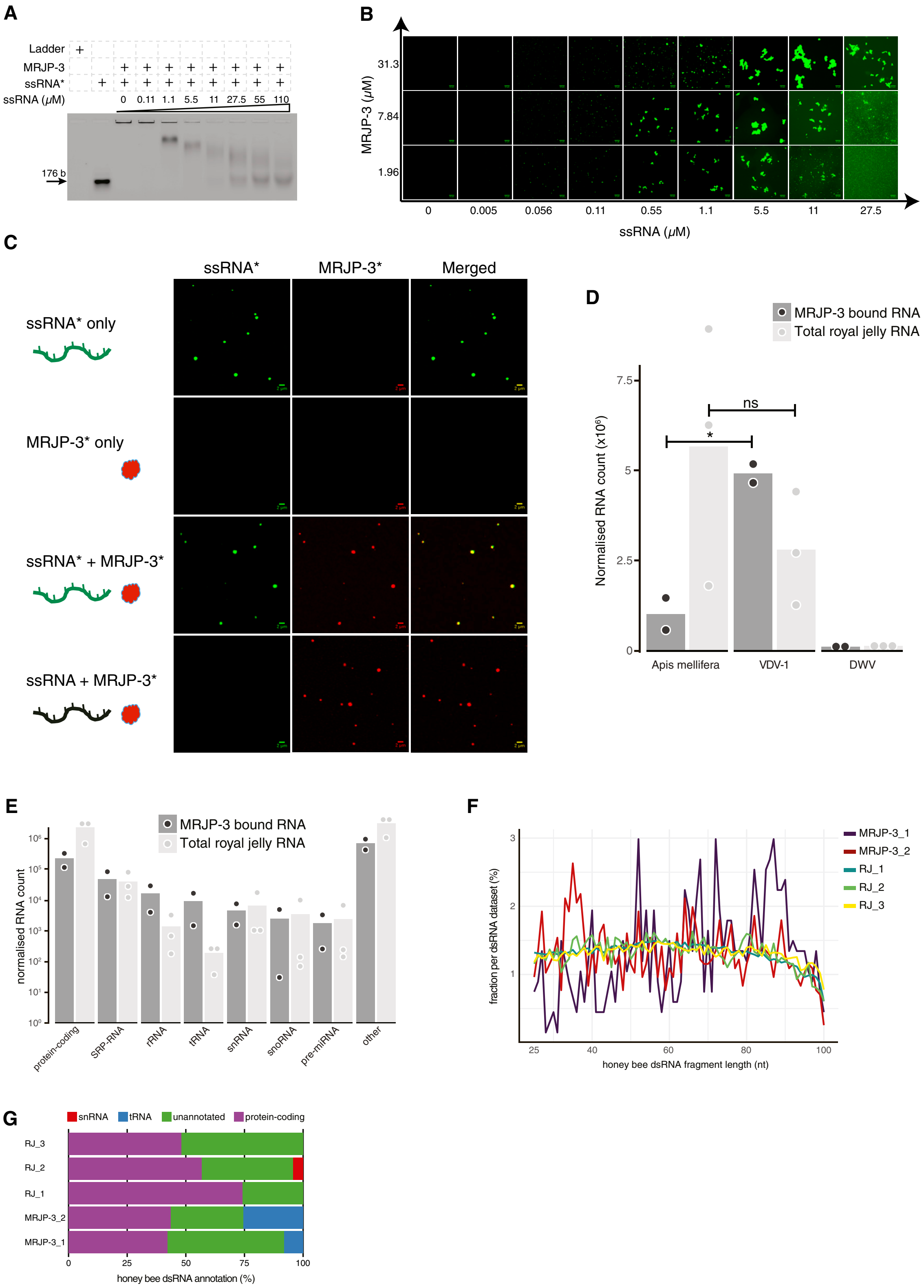

Figure 3. Multivalent RNA Binding Stimulates Super-order Assembly of Dynamic MRJP-3 RNPs and Isolation of RJ RNA Partners of MRJP-3

(A) The multivalent interaction of MRJP-3 with RNA is reversible. MRJP-3-bound ssRNA* was introduced to increasing quantities of unlabeled ssRNA. ssRNA* (0.04 µM) and MRJP-3 (31.3 µM) were used in all ssRNA*- and protein-containing treatments. Unlabeled DNA ladder served as labeling control.

(legend continued on next page)

RNA (Figure S3C), which is consistent with MRJP-3 binding RNA non-specifically. RNA sequencing (RNA-seq) revealed that MRJP-3 bound both endogenous (*Apis mellifera*) and exogenous (e.g., viral) RJ RNA (Figure 3D). MRJP-3 bound a higher proportion of Varroa destructor virus 1 (VDV-1) RNA than is present in total RJ RNA (Figure 3D). We observed full genome VDV-1 reads coverage, indicating that specific VDV-1 RNA fragments were not skewing the sequencing outcome (Figure S3D). MRJP-3 was not associated with specific honeybee RNAs and bound diverse protein- and non-coding RNAs (Figure 3E; Table S2).

We previously demonstrated that bees transmit biologically active dsRNA, and diverse complementary viral RNA fragments occur in worker and royal jellies (Maori et al., 2019). Consistently, MRJP-3 bound matching sense and antisense VDV-1 RNAs (Figure S3D) as well as honeybee pre-miRNA (microRNA) hairpins (Figure 3E). We screened for endogenous duplexed RNA and identified putative honeybee dsRNAs with a broad size distribution that somewhat vary between total and MRJP-3-bound RNA (Figure 3F). The majority of putative endogenous dsRNA derived from unannotated and protein-coding genes. However, MRJP-3 was associated with base-paired RNA fragments derived from tRNA genes (Figure 3G; Table S3). As base-pairing within the tRNA molecule involves 4–7 nt, and our dsRNA detection require at least 25 base-pairing nucleotides (Figure S3E), the data suggest that MRJP-3 binds duplexed tRNA fragments that are derived from two distinct RNA molecules. Our sequence analysis indicates that MRJP-3 binds diverse ssRNA and dsRNA populations and may show some specificity for VDV-1 RNA.

MRJP-3 Enhances Environmental RNA Stability and Uptake

Environmental RNA persistence requires RNA stabilization. MRJP-3 RNPs could potentially function to protect RJ RNA from factors such as nucleases. To test this, we mixed ssRNA with MRJP-3 or MRJP-1 and then introduced RNase-A. MRJP-3-bound RNA is protected from RNase-A degradation, whereas MRJP-1, which lacks the TRR domain, neither bound RNA nor protected it from degradation (Figure 4A). This experiment further demonstrated that the TRR is required for RNP assembly and that RNA binding facilitates nuclease protection. MRJP-3 binding also protects dsRNA from RNase-A degradation (Figure S4A). We imaged ssRNA-MRJP-3 RNPs in the presence or absence of RNase-A and did not observe substantial difference (Figure 4B). However, MRJP-3 binding does not prevent dsRNA processing by the Dicer-like RNase-III nuclease, suggesting that RNA within the MRJP-3 granules could be available for intracellular RNA-interacting factors (Figures S4B and S4C).

In the absence of suitable genetics, we could not directly determine whether MRJP-3 enhances ingested RNA uptake in honeybees. Instead, we investigated MRJP-3 function in a heterologous *C. elegans* nematode system. *C. elegans* is susceptible to environmental RNAi through ingestion (Timmons and Fire, 1998), and its genome does not encode MRJP-3 (Figure 2A). In *C. elegans*, UNC-22 is required for normal muscle morphology and physiology, and RNAi targeting *unc-22* mRNA provides a quantitative uncoordinated (UNC) phenotype (Fire et al., 1998). Therefore, we used *unc-22* RNAi system to test whether MRJP-3 affected environmental RNAi uptake in *C. elegans*. Animals fed on a bacterial diet supplemented with MRJP-3 bound to *unc-22* dsRNA (dsRNA-*unc-22*), dsRNA-*unc-22* alone, dsRNA-*unc-22* mixed with MRJP-1, or MRJP-3 bound to a control dsRNA (dsRNA-Fluc) (Figure 4C). Animals that were fed on MRJP-3-bound dsRNA-*unc-22* showed enhanced UNC phenotype compared with controls.

To test whether MRJP-3 protects dsRNA or also enhances dsRNA delivery in *C. elegans*, we designed an imaging experiment to detect dsRNA uptake shortly after ingestion. Animals were soaked for 2 h with labeled dsRNA alone, and in the presence of MRJP-3 or MRJP-1, followed by fixation and imaging (Figure 4D). Enhanced dsRNA uptake was observed in animals that ingested MRJP-3 RNPs. In *C. elegans*, dsRNA longer than 50 bp is taken up from the lumen into intestinal cells (McEwan et al., 2012). Thus, RNA degradation might hinder dsRNA uptake. To assess if MRJP-3's enhancement of RNAi might be due solely to dsRNA degradation in other treatments, we sampled soaking solutions prior to fixation and analyzed RNA integrity (Figure S4D). Although RNA was more stable when bound to MRJP-3, no substantial degradation occurred in any condition. We conclude that MRJP-3 RNPs actively enhances dsRNA uptake in *C. elegans*.

DISCUSSION

With the aim to uncover the mechanisms and factors that facilitate horizontal RNA transfer between honeybees, we have revealed a secreted RNA-aggregating jelly protein. MRJP-3 binds 18 nt and longer ssRNA and dsRNA in a non-sequence-specific

(B) MRJP-3 RNPs are affected by the protein/ssRNA mole ratio. Images of RNPs formed at various mole ratios of MRJP-3 and Alexa Fluor-488 labeled ssRNA (ssRNA*). Scale bar represents 10 μm.
(C) RNA mediates super-order assembly of MRJP-3 oligomers, resulting in RNP formation in soluble RJ fraction. RJ buffer or 4.28 μM Alexa Fluor-633 labeled MRJP-3 (MRJP-3*) was introduced to 50% soluble RJ fraction. ssRNA (0.15 μM) or ssRNA* was used in RNA-containing treatments. Scale bar represents 2 μm.
(D) MRJP-3 binds both endogenous (*Apis mellifera*) and exogenous (viral) RNA. Only viruses with a mapped fraction of at least 1% are shown. Points are individual biological replicates. Bars represent the mean across replicates. Horizontal lines indicate tests for significant enrichment of viral RNA over bee RNA in the MRJP-3 bound fraction, but not in RJ (*$p < 0.05$, two-sided t test).
(E) MRJP-3 is not associated with specific honeybee RNA species. Plots show reads from RNA-seq mapping to *Apis mellifera*. Only RNA types with a mapped fraction of at least 1% are shown. "Other" represents reads mapped to unannotated regions. Points are individual biological replicates. Bars represent the mean across replicates.
(F) Detection and size distribution of putative total and MRJP-3-bound RJ dsRNAs that are mapped to the *Apis mellifera* genome. dsRNA is detected when two distinct RNA molecules had at least 25 nt base pairs and the overhang on either side did not exceed 100 nt.
(G) MRJP-3 binds diverse putative endogenous dsRNA and is associated with duplexed tRNA fragments. Only RNA types with a mapped fraction of at least 1% are shown.
See also Figure S3 and Tables S2 and S3.

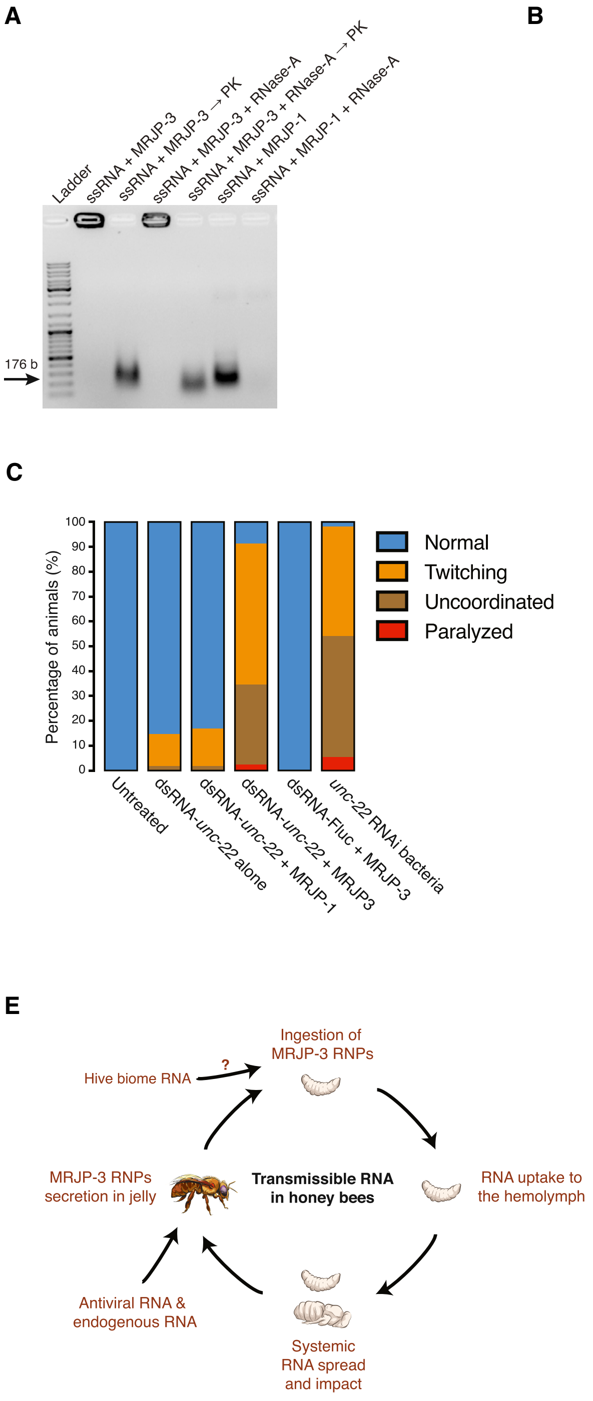

Figure 4. MRJP-3 RNP Granules Protect RNA From Degradation and Enhance RNA Bioavailability

(A) MRJP-3-bound RNA is protected from RNase-A digestion. Treatments included ssRNA mixed with MRJP-3, ssRNA mixed with MRJP-3 followed by incubation with PK, ssRNA mixed with MRJP-3 and RNase-A, ssRNA mixed with MRJP-3 and RNase-A followed by incubation with PK, ssRNA mixed with MRJP-1, and ssRNA mixed with MRJP-1 and RNase-A. ssRNA (0.3 μM) and MRJP-3 or MRJP-1 (42.8 μM) were used in all ssRNA- and protein-containing treatments. RNase challenge was performed by introducing 5 μg RNase-A followed by 1 h incubation at room temperature.

(B) RNase-A presence does not affect MRJP-3 RNPs. Images of RNPs formed with ssRNA* with or without RNase-A. ssRNA* (0.3 μM) and MRJP-3 or MRJP-1 (42.8 μM) were used in all ssRNA*- and protein-containing treatments. RNase challenge was performed by introducing 5 μg RNase-A followed by 1–3 h incubation at room temperature. Scale bar represents 2 μm.

(C) dsRNA-MRJP-3 RNPs enhance *unc-22* RNAi phenotype in *C. elegans*. Each treatment contained three biological repeats (n = 150 animals per treatment).

(legend continued on next page)

manner. The protein is assembled into a higher order oligomeric form in an RNA-independent manner. Multivalent RNA binding drives super-order assembly of MRJP-3 oligomers into eRNP granules. These granules concentrate and stabilize RNA and enhance its uptake by ingestion. Thus, MRJP-3 is a dietary factor that likely mediates horizontal RNA flow among honeybees (Figure 4E).

We have shown previously that honeybees are able to share biologically active dsRNA among individuals and generations in the hive (Maori et al., 2019). RNA transfer is mediated by secretion and consumption of jelly, which is highly enriched for MRJP-3. The transmission of RNA could drive social immunity against the Varroa mite (Garbian et al., 2012) and presumably against other pathogens such as viruses (Maori et al., 2019). Consistently, sense and antisense viral RNA fragments represent a high proportion of the natural substrates of MRJP-3 (Figure S3D). Furthermore, diverse putative dsRNAs, which are mapped to the honeybee genome, occur in worker and royal jellies and bound by MRJP-3 (Figures 3F and 3G; Maori et al., 2019). Notably, MRJP-3 is associated with tRNA fragments, which have been shown to inhibit retrotransposons activity and regulate epigenetic inheritance of metabolic traits (Chen et al., 2016; Schorn et al., 2017). The multivalent interaction of MRJP-3 and its RNA substrates is somewhat analogous to the polyclonal antibody-antigen precipitation dynamics (Heidelberger and Kendall, 1935). Thus, MRJP-3 might act as a factor that sponges environmental RNA reservoirs for downstream detection and processing by RNA receptors or effectors, such as Dicer. The presence of MRJP-3 in the hemolymph (Chan et al., 2009; Randolt et al., 2008), and the susceptibility of MRJP-3 RNPs to RNase-III digestion (in contrast to their RNase-A resistance), supports the involvement of MRJP-3 eRNPs in mediating ingested RNA bioavailability (Figures 4C and 4D). Further research is required to elucidate the mechanism of RNA uptake mediated by MRJP-3 eRNPs as well as its physiological roles at the individual and colony levels.

Assembly of membrane-less RNP organelles, such as stress granules and P bodies, involves liquid-liquid phase separation (Hyman et al., 2014). RNP compartments can be formed by another phase-transition mechanism, in which protein self-polymerization aggregates into an RNA-recruiting scaffold, as described for Xvelo in the Balbiani bodies (Boke et al., 2016). Yet these forms of RNA-scaffolded multi-protein assembly have been identified only within cells. We suggest that macromolecular RNP assemblies may also occur outside the cell and the organism. Our data show that RNA interconnects MRJP-3 oligomers into an RNP condensate; hence, while self-polymerization could explain the formation of defined MRJP-3 oligomers, it does not exclusively drive the phase transition and formation of MRJP-3 eRNP granules.

Cellular RNP compartments are dynamic in size and content because of the continuous exchange of material with their surroundings (Hyman et al., 2014). MRJP-3 eRNPs are dynamic as well, and the RNA/MRJP-3 ratio affects their size, similar to other prion-like proteins that phase-separate upon RNA binding (Maharana et al., 2018). MRJP-3 binds a diverse RNA population in a non-sequence-specific manner. However, binding is constrained to RNAs that are 18 nt or longer. Therefore, MRJP-3 substrates include nucleic acids that differ in length and structure complexity, potentially affecting the eRNP granule properties. Inside cells, RNP organelles are thought to facilitate specific chemical and enzymatic reactions that are essential for cell viability. Here we demonstrated that MRJP-3 eRNP granules could function to shield RNA from hostile environmental factors and to enhance RNA uptake. We speculate that high-order eRNP assemblies play diverse roles within and outside the organism.

STAR★METHODS

Detailed methods are provided in the online version of this paper and include the following:

- KEY RESOURCES TABLE
- CONTACT FOR REAGENT AND RESOURCE SHARING
- EXPERIMENTAL MODEL AND SUBJECT DETAILS
  - Reproductive hive system
  - Nematode culture
- METHOD DETAILS
  - Detection of Alexa Fluor-488 labeled dsRNA in royal jelly
  - Immunohistochemistry
  - Royal and worker jelly samples
  - dsRNA and ssRNA synthesis
  - RNA labeling
  - Electrophoretic Mobility Shift Assays (EMSA)
  - Extraction of soluble fraction of RJ
  - RNA extraction from royal jelly
  - RT-PCR
  - Protein disorder prediction
  - Taxonomic tree
  - Isolation of proteins from royal jelly
  - Recombinant MRJP-3 expression and purification
  - Microscale thermophoresis
  - Size exclusion chromatography
  - Microscopy
  - Super-resolution imaging
  - Protein biotinylation
  - RJ RNA pull down
  - *C. elegans* soaking
  - *C. elegans* unc-22 RNAi

(D) MRJP-3 RNPs enhances ingested dsRNA uptake in *C. elegans*. Animals were soaked in the presence of MRJP-3 RNPs formed with Alexa Fluor-647-labeled dsRNA-Fluc (dsRNA**). Control groups included soaking animals with dsRNA** mixed with MRJP-1, dsRNA** mixed in RJ buffer, and MRJP-3 alone. dsRNA** (2.15 nM) and MRJP-3 or MRJP-1 (42.8 μM) were used in all dsRNA- and protein-containing treatments.
(E) A working model describing the role of MRJP-3 in the transmissible RNA pathway in honeybees. Nurse bees secrete jelly-containing RNPs that comprise endogenous and exogenous (e.g., viral, fungi, bacteria, plant) RNAs. Bee larvae ingest environmental MRJP-3 RNPs through jelly consumption. The ingested RNA is taken up to the hemolymph, is systemically spread, and affects gene expression including an antiviral response.
See also Figure S4.

- Mass spectrometry of royal and worker jelly samples
- RNA library preparation and sequencing

- QUANTIFICATION AND STATISTICAL ANALYSIS
  - RNA-seq analysis
  - Viral coverage
  - GO analysis
  - MicroRNA screen
  - Double-stranded honeybee RNA screen
- DATA AND SOFTWARE AVAILABILITY

SUPPLEMENTAL INFORMATION

Supplemental Information can be found online at https://doi.org/10.1016/j.molcel.2019.03.010.

ACKNOWLEDGMENTS

We thank John Rayner (Springwell Apiaries) for providing bee and jelly samples. We thank the Heeney and Miska groups (Cambridge University) for their input on the study; Peter Lachmann, Laurence Tiley, and Shahar Avin (Cambridge University) for inspiring discussions and advice; and Nicola Lawrence and Richard Butler (Imaging Facility, Gurdon Institute) for their support on the imaging. This work was supported by Cancer Research UK (C13474/A18583 and C6946/A14492) and the Wellcome Trust (104640/Z/14/Z and 092096/Z/10/Z) to K.L.M.R, A.S., and E.A.M.; Cancer Research UK (C57233/A22356) to C.-C.L. and J.E.L.; Science without Borders doctorate scholarship (205589/2014-6; CNPq, Brazilian Federal Government) to I.C.N.; a Marie Curie Intra-European Fellowship for Career Development (PIEF-GA-2010-274406); and a Leo Baeck Scholarship and Herchel Smith Postdoctoral Fellowship (XXACC_AFGLTRB2) to E.M.

AUTHOR CONTRIBUTIONS

E.M. conceptualized and supervised the study. E.M., J.L.H., and E.A.M. designed the experiments. E.M. performed the biochemistry and imaging experiments. H.B. and J.D.E. performed the beehive experiments. D.J.S. and E.M. purified native MRJP-3 and MRJP-1. I.C.N. and E.M. performed the *C. elegans* experiments. A.S. and K.L.M.R. generated the RNA-seq libraries and analyzed the sequencing data. C.-C.L. performed the MST and gel filtration experiments. E.M., J.E.L., J.L.H., and E.A.M. analyzed the experiments. E.M. and E.A.M. wrote the paper with input from all authors.

DECLARATION OF INTERESTS

The authors declare no competing interests. E.M. is a founder and director of Tropic Biosciences. E.A.M. is a founder and director of STORM Therapeutics. Tropic Biosciences and STORM Therapeutics had no role in the design of the study and collection, analysis, and interpretation of data or in writing the manuscript.

Received: July 3, 2018
Revised: December 10, 2018
Accepted: March 5, 2019
Published: May 2, 2019

REFERENCES

Albertová, V., Su, S., Brockmann, A., Gadau, J., and Albert, S. (2005). Organization and potential function of the mrjp3 locus in four honeybee species. J. Agric. Food Chem. *53*, 8075–8081.

Anders, S., and Huber, W. (2010). Differential expression analysis for sequence count data. Genome Biol. *11*, R106.

Boke, E., Ruer, M., Wühr, M., Coughlin, M., Lemaitre, R., Gygi, S.P., Alberti, S., Drechsel, D., Hyman, A.A., and Mitchison, T.J. (2016). Amyloid-like self-assembly of a cellular compartment. Cell *166*, 637–650.

Brenner, S. (1974). The genetics of Caenorhabditis elegans. Genetics *77*, 71–94.

Buck, A.H., Coakley, G., Simbari, F., McSorley, H.J., Quintana, J.F., Le Bihan, T., Kumar, S., Abreu-Goodger, C., Lear, M., Harcus, Y., et al. (2014). Exosomes secreted by nematode parasites transfer small RNAs to mammalian cells and modulate innate immunity. Nat. Commun. *5*, 5488.

Cai, Q., Qiao, L., Wang, M., He, B., Lin, F.-M., Palmquist, J., Huang, S.D., and Jin, H. (2018). Plants send small RNAs in extracellular vesicles to fungal pathogen to silence virulence genes. Science *360*, 1126–1129.

Chan, Q.W.T., Melathopoulos, A.P., Pernal, S.F., and Foster, L.J. (2009). The innate immune and systemic response in honey bees to a bacterial pathogen, Paenibacillus larvae. BMC Genomics *10*, 387.

Chen, Q., Yan, M., Cao, Z., Li, X., Zhang, Y., Shi, J., Feng, G.-H., Peng, H., Zhang, X., Zhang, Y., et al. (2016). Sperm tsRNAs contribute to intergenerational inheritance of an acquired metabolic disorder. Science *351*, 397–400.

Dobin, A., Davis, C.A., Schlesinger, F., Drenkow, J., Zaleski, C., Jha, S., Batut, P., Chaisson, M., and Gingeras, T.R. (2013). STAR: ultrafast universal RNA-seq aligner. Bioinformatics *29*, 15–21.

Federhen, S. (2012). The NCBI Taxonomy database. Nucleic Acids Res. *40*, D136–D143.

Fire, A., Xu, S., Montgomery, M.K., Kostas, S.A., Driver, S.E., and Mello, C.C. (1998). Potent and specific genetic interference by double-stranded RNA in Caenorhabditis elegans. Nature *391*, 806–811.

Furusawa, T., Rakwal, R., Nam, H.W., Shibato, J., Agrawal, G.K., Kim, Y.S., Ogawa, Y., Yoshida, Y., Kouzuma, Y., Masuo, Y., and Yonekura, M. (2008). Comprehensive royal jelly (RJ) proteomics using one- and two-dimensional proteomics platforms reveals novel RJ proteins and potential phospho/glycoproteins. J. Proteome Res. *7*, 3194–3229.

Garbian, Y., Maori, E., Kalev, H., Shafir, S., and Sela, I. (2012). Bidirectional transfer of RNAi between honey bee and Varroa destructor: Varroa gene silencing reduces Varroa population. PLoS Pathog. *8*, e1003035.

Halfmann, R., Alberti, S., Krishnan, R., Lyle, N., O'Donnell, C.W., King, O.D., Berger, B., Pappu, R.V., and Lindquist, S. (2011). Opposing effects of glutamine and asparagine govern prion formation by intrinsically disordered proteins. Mol. Cell *43*, 72–84.

Heidelberger, M., and Kendall, F.E. (1935). The precipitin reaction between type III pneumococcus polysaccharide and homologous antibody: III. A quantitative study and a theory of the reaction mechanism. J. Exp. Med. *61*, 563–591.

Hunter, W., Ellis, J., Vanengelsdorp, D., Hayes, J., Westervelt, D., Glick, E., Williams, M., Sela, I., Maori, E., Pettis, J., et al. (2010). Large-scale field application of RNAi technology reducing Israeli acute paralysis virus disease in honey bees (Apis mellifera, Hymenoptera: Apidae). PLoS Pathog. *6*, e1001160.

Hyman, A.A., Weber, C.A., and Jülicher, F. (2014). Liquid-liquid phase separation in biology. Annu. Rev. Cell Dev. Biol. *30*, 39–58.

Kovacs, E., Tompa, P., Liliom, K., and Kalmar, L. (2010). Dual coding in alternative reading frames correlates with intrinsic protein disorder. Proc. Natl. Acad. Sci. U S A *107*, 5429–5434.

Li, H., Handsaker, B., Wysoker, A., Fennell, T., Ruan, J., Homer, N., Marth, G., Abecasis, G., and Durbin, R.; 1000 Genome Project Data Processing Subgroup (2009). The sequence alignment/map format and SAMtools. Bioinformatics *25*, 2078–2079.

Liao, Y., Smyth, G.K., and Shi, W. (2013). The Subread aligner: fast, accurate and scalable read mapping by seed-and-vote. Nucleic Acids Res. *41*, e108.

Lin, Y., Protter, D.S.W., Rosen, M.K., and Parker, R. (2015). Formation and maturation of phase-separated liquid droplets by RNA-binding proteins. Mol. Cell *60*, 208–219.

Maharana, S., Wang, J., Papadopoulos, D.K., Richter, D., Pozniakovsky, A., Poser, I., Bickle, M., Rizk, S., Guillén-Boixet, J., Franzmann, T.M., et al. (2018). RNA buffers the phase separation behavior of prion-like RNA binding proteins. Science *360*, 918–921.

Maori, E., Lavi, S., Mozes-Koch, R., Gantman, Y., Peretz, Y., Edelbaum, O., Tanne, E., and Sela, I. (2007). Isolation and characterization of Israeli acute

paralysis virus, a dicistrovirus affecting honeybees in Israel: evidence for diversity due to intra- and inter-species recombination. J. Gen. Virol. *88*, 3428–3438.

Maori, E., Paldi, N., Shafir, S., Kalev, H., Tsur, E., Glick, E., and Sela, I. (2009). IAPV, a bee-affecting virus associated with Colony Collapse Disorder can be silenced by dsRNA ingestion. Insect Mol. Biol. *18*, 55–60.

Maori, E., Garbian, Y., Kunik, V., Mozes-Koch, R., Malka, O., Kalev, H., Sabath, N., Sela, I., and Shafir, S. (2019). A Transmissible RNA Pathway in Honey Bees. Cell Rep. *27*, https://doi.org/10.1016/j.celrep.2019.04.073.

Martin, M. (2011). Cutadapt removes adapter sequences from high-throughput sequencing reads. EMBnet.journal *17*, 10–12.

McEwan, D.L., Weisman, A.S., and Hunter, C.P. (2012). Uptake of extracellular double-stranded RNA by SID-2. Mol. Cell *47*, 746–754.

McMenamin, A.J., and Genersch, E. (2015). Honey bee colony losses and associated viruses. Curr. Opin. Insect Sci. *8*, 121–129.

Michelitsch, M.D., and Weissman, J.S. (2000). A census of glutamine/asparagine-rich regions: implications for their conserved function and the prediction of novel prions. Proc. Natl. Acad. Sci. U S A *97*, 11910–11915.

Molnar, A., Melnyk, C.W., Bassett, A., Hardcastle, T.J., Dunn, R., and Baulcombe, D.C. (2010). Small silencing RNAs in plants are mobile and direct epigenetic modification in recipient cells. Science *328*, 872–875.

Okamoto, I., Taniguchi, Y., Kunikata, T., Kohno, K., Iwaki, K., Ikeda, M., and Kurimoto, M. (2003). Major royal jelly protein 3 modulates immune responses in vitro and in vivo. Life Sci. *73*, 2029–2045.

Quinlan, A.R., and Hall, I.M. (2010). BEDTools: a flexible suite of utilities for comparing genomic features. Bioinformatics *26*, 841–842.

R Development Core Team (2018). R: A language and environment for statistical computing (Vienna, Austria: R Foundation for Statistical Computing). http://www.R-project.org/.

Randolt, K., Gimple, O., Geissendörfer, J., Reinders, J., Prusko, C., Mueller, M.J., Albert, S., Tautz, J., and Beier, H. (2008). Immune-related proteins induced in the hemolymph after aseptic and septic injury differ in honey bee worker larvae and adults. Arch. Insect Biochem. Physiol. *69*, 155–167.

Scharlaken, B., de Graaf, D.C., Goossens, K., Peelman, L.J., and Jacobs, F.J. (2008). Differential gene expression in the honeybee head after a bacterial challenge. Dev. Comp. Immunol. *32*, 883–889.

Schorn, A.J., Gutbrod, M.J., LeBlanc, C., and Martienssen, R. (2017). LTR-retrotransposon control by tRNA-derived small RNAs. Cell *170*, 61–71.e11.

Shahid, S., Kim, G., Johnson, N.R., Wafula, E., Wang, F., Coruh, C., Bernal-Galeano, V., Phifer, T., dePamphilis, C.W., Westwood, J.H., and Axtell, M.J. (2018). MicroRNAs from the parasitic plant Cuscuta campestris target host messenger RNAs. Nature *553*, 82–85.

Stocker, A., Schramel, P., Kettrup, A., and Bengsch, E. (2005). Trace and mineral elements in royal jelly and homeostatic effects. J. Trace Elem. Med. Biol. *19*, 183–189.

Tamura, S., Amano, S., Kono, T., Kondoh, J., Yamaguchi, K., Kobayashi, S., Ayabe, T., and Moriyama, T. (2009). Molecular characteristics and physiological functions of major royal jelly protein 1 oligomer. Proteomics *9*, 5534–5543.

Timmons, L., and Fire, A. (1998). Specific interference by ingested dsRNA. Nature *395*, 854.

Winston, W.M., Molodowitch, C., and Hunter, C.P. (2002). Systemic RNAi in C. elegans requires the putative transmembrane protein SID-1. Science *295*, 2456–2459.

Wright, G.A., Nicolson, S.W., and Shafir, S. (2018). Nutritional physiology and ecology of honey bees. Annu. Rev. Entomol. *63*, 327–344.

Young, M.D., Wakefield, M.J., Smyth, G.K., and Oshlack, A. (2010). Gene ontology analysis for RNA-seq: accounting for selection bias. Genome Biol. *11*, R14.

Zhu, K., Liu, M., Fu, Z., Zhou, Z., Kong, Y., Liang, H., Lin, Z., Luo, J., Zheng, H., Wan, P., et al. (2017). Plant microRNAs in larval food regulate honeybee caste development. PLoS Genet. *13*, e1006946.

STAR★METHODS

KEY RESOURCES TABLE

REAGENT or RESOURCE	SOURCE	IDENTIFIER
Antibodies		
Rabbit polyclonal anti-Alexa Fluor-488	Thermo Fisher Scientific	Cat# A-11094; RRID: AB_221544
Bacterial and Virus Strains		
Escherichia coli HB101 strain	*Caenorhabditis* Genetics Center (CGC)	HB101 strain
E. coli HT115 (DE3) RNAi strain - empty vector	Julie Ahringer lab	N/A
E. coli HT115 (DE3) RNAi strain - *unc-22* (*ZK617.1*)	Julie Ahringer lab	N/A
Biological Samples		
Commercial raw RJ	Well Bee-ing UK	50 gr pure fresh royal jelly: https://www.royaljellyinhoney.co.uk/buy-royal-jelly.html
Chemicals, Peptides, and Recombinant Proteins		
Recombinant full-length MRJP-3	This paper	N/A
Recombinant truncated MRJP-3	This paper	N/A
Deposited Data		
Raw RNA-seq data	This paper	ArrayExpress: E-MTAB-6732
Raw imaging data	This paper	Mendeley Data: https://doi.org/10.17632/5w7rbd8452.1
Experimental Models: Cell Lines		
Sf9 (insect; *Spodoptera frugiperda*)	AATC	ATCC Cat# CRL-1711; RRID: CVCL_0549
Experimental Models: Organisms/Strains		
Caenorhabditis elegans wild type strain	*Caenorhabditis* Genetics Center (CGC)	Wild type strain N2 (var Bristol)
Oligonucleotides		
List and sequences of RNA oligos Table S4A)	This paper	N/A
List and sequences of primers (Table S4B)	This paper	N/A
Recombinant DNA		
Baculovirus expression vector pVL1393	Expression Systems	Cat# 91-013
Software and Algorithms		
RNA-seq analysis scripts	This paper	https://doi.org/10.5281/zenodo.1542860
GNU R 3.4.4	R Development Core Team (2018)	https://www.r-project.org/
STAR 5.2.5b	Dobin et al., 2013	https://github.com/alexdobin/STAR
samtools	Li et al., 2009	https://github.com/samtools
cutadapt 1.11	Martin, 2011	https://cutadapt.readthedocs.io/en/stable/installation.html
bedtools 2.27.1	Quinlan and Hall, 2010	https://github.com/arq5x/bedtools2/releases/tag/v2.27.1
Subread featureCounts 1.5.0-p2	Liao et al., 2013	http://subread.sourceforge.net/
goseq 1.28	Young et al., 2010	https://bioconductor.org/packages/release/bioc/html/goseq.html
Other		
Labeled ribonucleotide Alexa Fluor 488-5-UTP	Thermo Fisher Scientific	Cat# C11403
EnvisionTM Flex kit system	Agilent	Cat# K802421-2
MEGAscript T7 Transcription Kit	Thermo Fisher Scientific	Cat# AM1334
Ulysis Alexa Fluor 488/647 Nucleic Acid Labeling Kits	Thermo Fisher Scientific	Cat# U21650
		Cat# U21660
TruSeq Stranded Total RNA Library Prep kit	Illumina	Cat# 20020596
ULTRA BEE pollen substitute	ManLake	FD-374

CONTACT FOR REAGENT AND RESOURCE SHARING

Further information and requests for resources and reagents should be directed to the lead contacts, Eyal Maori (eyalmm@gmail.com) and Eric Miska (eam29@cam.ac.uk).

EXPERIMENTAL MODEL AND SUBJECT DETAILS

Reproductive hive system

Caged fertile queen bees, together with approximately 1000 worker bees, were placed in standard 5-frame wooden nuc box with separate bottom board. The hives were sealed for three days in which the combs were constructed and queen-workers recognition had been established. During the first three days, the bees were fed on a mixture of 33% honey and 67% sucrose powder (candy). Next, the hives were transferred into two net-houses separating between dsRNA treated, and untreated hives. The bees were free to fly within the net-houses and to forage for water from buckets. The first 14 days were an adaptation period, during which the colonies were fed on demand with candy, and pollen supplement patties (10 g each) which were placed on top of the combs and replaced once a week. An established colony was determined by at least two constructed combs and egg-laying activity of the queen; only these hives were included in the experiment. During the experiments, established colonies (two per treatment) were fed on pollen supplement patties (10 g each), and had an unlimited water supply.

Nematode culture

C. elegans Bristol N2 strain was grown and maintained as previously described (Brenner, 1974). The nematodes were kept at 20°C, unless otherwise indicated. HB101 strain *E. coli* was used as a food source. For maintenance, animals were kept in nematode growth media (NGM) agar plates and transferred using a platinum wire under a dissecting microscope (Leica M50). Alternatively, pieces of one plate were chunked and placed facing down on a new plate.

METHOD DETAILS

Detection of Alexa Fluor-488 labeled dsRNA in royal jelly

50 mL 10% (v/w) sucrose solutions with or without Alexa Fluor-488 labeled dsRNA (dsRNA*, 4 ng/μl final concentration) were provided on days: 1, 2, 4 and 5 (two hives per treatment). On day-5, queens were removed to initiate queen rearing and RJ secretion. On day-9, 3rd-4th instar larvae were carefully removed from queen brood cells with a fine paintbrush, and the larvae were checked to be intact without any physical damage. RJ was harvested from such queen brood cells and samples from each hive were pooled and stored at −80°C.

Immunohistochemistry

RJ samples were transferred into a cryomold followed by a frozen section media treatment (Leica, FSC, 22 clear). 10μM sections were cut by a cryostat (Leica, CM1900), put on slides and left at room temperature to dry. Slides were fixed for 15 min in 4% PFA (in PBS) and washed twice for 5min with 1xPBS. Immunostaining was performed by the Dako Autostainer Link 48 with the Envision Flex kit system (Dako) according to the manufacturer's instructions using 1:250 diluted Alexa Fluor-488 antibody (Thermo Fisher, Cat. A-11094). More specifically, sections were incubated for 10 min with peroxidase-blocking reagent, 60 min with 1:250 diluted primary polyclonal rabbit anti-Alexa Fluor-488, 30 min with the EnVision FLEX/HRP Detection Reagent, 5 min with EnVision FLEX DAB+ Chromogen/EnVision FLEX Substrate Buffer mix, and 5 min with EnVision FLEX Hematoxylin. The slides were then dehydrated (3 min in 70% ethanol, 3 min in 95% ethanol and 3 min in 100% ethanol) followed by 2 times 5 min wash in xylene and then mounted.

Royal and worker jelly samples

Royal and worker jellies, which were directly applied in experiments (raw or soluble fraction), were produced in collaboration with Springwell Apiaries, UK. RJ was harvested from brood cells containing 3rd-5th instar queen larvae. Worker jelly was collected from brood cells with 4th-5th instar worker larvae by washing cells with nuclease-free water to resuspend the low jelly quantity available. Prior to jelly harvest, larvae were carefully removed and checked for any physical damage. Commercial RJ was sourced from Well Bee-ing UK and was used for MRJP-3 isolation.

dsRNA and ssRNA synthesis

dsRNA and ssRNA that are longer than 50 nt were synthesized by *in vitro* transcription using Megascript kit (Ambion) according to the manufacturer's instructions including DNase-I treatment. Transcription DNA templates, carrying a single (for ssRNA synthesis) or double opposite T7 promoters (for dsRNA synthesis), were generated by PCR or gene synthesis. HPLC-purified RNA oligos (up to 50 nt) were ordered from Integrated DNA Technologies (IDT). List of nucleic acid sequences, their corresponding NCBI accession number and source is shared in Table S4.

RNA labeling

Long ssRNA/dsRNA (> 50 nt) was labeled by *in vitro* transcription reaction using Alexa Fluor-488 labeled UTP (Thermo Scientific). 2 μl of 1mM labeled UTP was added to a standard 20 μl *in vitro* transcription reaction of the Megascript kit (Ambion). Ulysis kit (Thermo Scientific) was used according to the manufacturer's instructions to label RNA oligos (50 nt and shorter) with Alexa Fluor-488 as well as the Alexa Fluor-647 labeled dsRNA.

Electrophoretic Mobility Shift Assays (EMSA)

Due to the large size of RNA-MRJP-3 complexes, non-denaturing agarose gel was used for the EMSA assays. Prior to the electrophoresis, RNA and protein samples (purified protein or dialysed eluted fraction) were mixed and incubated at room temperature for 0.5-3 hours, and then mixed with 1x loading buffer (Thermo Fisher Scientific, catalog no. R0611). Samples were loaded in Ethidium Bromide containing 0.8% non-denaturing agarose gel and run in 1xTAE buffer for 45 min in 150 mA.

Extraction of soluble fraction of RJ

Soluble RJ fraction was prepared by diluting raw RJ with "RJ buffer" (v/v) that was formulated based on RJ ash content (Stocker et al., 2005): 77 mM KCl / 10 mM MgCl2 / 44 mM NaCl / 2.5 mM CaCl2 / 30 mM Acetate pH 4.0. The diluted RJ was then centrifuged at 16 K *rcf.* for 10 min at room temperature and the aqueous fraction was collected. The centrifugation and aqueous fraction separation were performed three additional times until a pure soluble fraction was extracted.

RNA extraction from royal jelly

1 mL of 25% (v/v) RJ was split into 2 equal aliquots. RNA was purified separately from each aliquot by phenol/chloroform/isoamyl alcohol extraction and pooled together.

RT-PCR

Two-step primer-specific RT-PCR was performed by following standard SuperScript-III (invitrogen) and KAPA HiFi Hotstart ready mix PCR (Kapa Biosystems) protocols. Reverse transcription was performed by applying 0.5 ng total RJ RNA template, two dsRNA-specific primers (0.25 μM final concentration for each primer; sequences are detailed in Table S4). Same primers and 1 μl cDNA were applied in the PCR.

Protein disorder prediction

Intrinsic disorder in MRJP-3 (UniProt ID Q17060) was predicted by PONDR VSL2 (http://www.pondr.com/) and IUPred (http://iupred.enzim.hu/), which apply different approaches for disorder prediction (Kovacs et al., 2010). The default cutoff value of 0.5 was used in both algorithms.

Taxonomic tree

MRJP-3 amino acid sequence (UniProt ID Q17060) was blasted against the available non-redundant protein sequence database (nr). The outcome was analyzed for the presence of the yellow-related protein and TRR (amino acids 1-419 and 424-523 respectively). Next, the NCBI taxonomy database was applied to generate a common tree (Federhen, 2012).

Isolation of proteins from royal jelly

MRJP-3 and MRJP-1 were isolated by FPLC using ion exchange chromatography followed by hydroxyapatite chromatography.

RJ sample preparation

50 mL 10% soluble RJ was prepared and dialyzed overnight in MES binding buffer (25 mM MES, 0.15 M, pH 6.0), centrifuged at 16,000 *rcf.* and passed through a 0.22 μM filter.

MRJP-3 purification

MRJP-3 was purified using cation exchange FPLC. The RJ sample was loaded onto a Source 15S column (10 × 98 mm; GE Healthcare, cat. 17094401 or 28406415), equilibrated with MES binding buffer (2 mL per min) followed by a 6-column volume wash with MES binding buffer. Elution was performed with a linear 38 column volume salt gradient, from 25 mM MES pH 6.0 / 0.15M NaCl (Buffer-A) to 25 mM MES pH 6.0 / 1.0 M NaCl (Buffer-B). The cation exchange included overall 10 unbound and 58 eluted fractions. 20 fractions (approximately 5 mL per fraction) were collected over the first third of the elution gradient and their protein concentration was measured by Nanodrop spectrophotometer. MRJP-3 eluted over the first 25% gradient. Fractions containing the protein peak were run on 4%–12% gradient SDS-PAGE gel and those with the most concentrated and pure MRJP-3 were pooled and dialyzed into CHAP binding buffer (5 mM phosphate pH6.8 / 0.15 M NaCl). MRJP-3 was further purified on a Ceramic Hydroxyaptatite (CHAP) column (10 × 108 mm, CHAP Type I Tricorn 10/100 column; Bio-Rad cat. 157-0020, GE healthcare cat. 28406415). MRJP-3 sample was passed through the column (2 mL per min) followed by washing with 6 column volumes of CHAP buffer. Elution was performed with a linear 25 column volume gradient, from 5 mM phosphate pH 6.8 / 0.15 M NaCl (Buffer-C) to 500 mM phosphate pH 6.8 / 0.15 M NaCl (Buffer-D). The hydroxyaptatite chromatography included overall 12 unbound and 70 eluted fractions. MRJP-3 eluted over the first 30% gradient and 20 fractions (approximately 3 mL per fraction) were collected. Fractions with the most concentrated and pure MRJP-3 (at least 95% purity; determined by protein gel electrophoresis on 4%–12% gradient SDS-PAGE) were pooled and dialysed

into RJ Buffer. MRJP-3 concentration was determined by absorbance at 280 nm value using a 1 mg/ml/cm extinction coefficient of 0.8.

MRJP-1 purification

Starting material was unbound protein in the run-through from the Source 15S column (in MES binding buffer) under the conditions for purifying MRJP-3. MRJP-1 was purified using anion exchange FPLC performed on Source 15Q Tricorn 10/100 column (10 × 98 mm; GE healthcare, cat. 17094720 or 28406415). The unbound protein sample was passed through the column (2 mL per min) followed by washing with 6 column volumes of MES buffer. Elution was performed with a linear 35-column volume gradient, from Buffer-A to Buffer-B. The anion exchange included overall 12 unbound and 54 eluted fractions. 30 fractions (approximately 5 mL per fraction) were collected over the first half of the elution gradient. Fractions containing the protein peak were run on 4%–12% gradient SDS-PAGE gel and those with the most concentrated and pure MRJP-1 were pooled and dialyzed into CHAP binding buffer. MRJP-1 was further purified on a CHAP column (10 × 108 mm, CHAP Type I Tricorn 10/100 column; Bio-Rad cat. 157-0020, GE healthcare cat. 28406415). The MRJP-1 sample was passed through the column (2 mL per min) followed by washing with 6 column volumes of CHAP buffer. Elution was performed with a linear 25-column volume gradient, from Buffer-C to Buffer-D. The hydroxyaptatite chromatography included overall 9 unbound and 53 eluted fractions. 20 fractions (approximately 4 mL per fraction) were collected over the first 40% of the elution gradient and MRJP-1 eluted over the first 30% gradient. Fractions with the most concentrated and pure MRJP-1 (at least 95% purity; determined by protein gel electrophoresis on gradient 4%–12% SDS-PAGE) were pooled and dialysed into RJ Buffer. MRJP-1 concentration was determined by absorbance at 280 nm value using a 1 mg/ml/cm extinction coefficient of 1.2. MRJP-1 and MRJP-3 aliquots were stored in $-80\ ^{0}c$ and purified protein identities were validated by a MALDI/MS peptide mass fingerprinting, performed by the Cambridge Centre for Proteomics.

Recombinant MRJP-3 expression and purification

Recombinant MRJP-3 expression was serviced from the Israel Structural Proteomics Centre, Weizmann Institute. Full-length MRJP-3 (1-544) and truncated MRJP-3 (1-424) were cloned into baculovirus expression vector pVL1393. T7-epitope and three amino-acid (aa) linker (SAG) followed by 6xHis were introduced into the gene following the authentic secretion signal (aa 1-20). Each expression vector construct was co-transfected with the ProGreen green fluorescent protein (GFP) linearized baculovirus DNA (AB vector) into Sf9 insect cells. Viruses were produced for each construct, and were used to infect Sf9 cells for protein expression. Infection efficiency was monitored by GFP fluorescence of infected cells. Cells were grown in ESF921 protein-free culture medium (Expression Systems). Three days post-infection, medium containing the secreted protein was collected concentrated and dialyzed against 50 mM Tris 8.0 / 100 mM NaCl. Protein was purified on HisTrap_FF_crude_5 mL column (GE Healthcare) followed by desalting column to exchange the buffer of the eluted protein. The protein was further purified by anion exchange at pH 8.0 on Tricorn Q 10/100 GL column (GE Healthcare) and eluted at 75 mM salt. The fractions containing the eluted protein were dialyzed against RJ buffer.

Microscale thermophoresis

The binding affinities were measured using the Monolith NT.115 (NanoTemper Technologies, GmbH). Both single- and double-stranded 22 nt RNA were fluorescently labeled with Alexa Fluor-488 using the Ulysis kit (Thermo Scientific) according to the manufacturer's protocol. Labeling efficiency was determined to be 1:1 (RNA to dye) by measuring the absorbance at 260 and 488 nm. A 16 step dilution series of the unlabeled MRJP-3 was prepared and mixed with the labeled RNA at 1:1 ratio and loaded into capillaries. Measurements were performed at 25°C in RJ buffer containing 0.01% Tween 20. Data analyses were performed using Nanotemper Analysis software, v.1.2.101 and were plotted using Origin 7.0. All measurements were conducted as triplicates and the errors were presented as the standard error of the triplicates.

Size exclusion chromatography

MRJP-3 was concentrated to 100 μM and the concentrated MRJP-3 was injected onto a 16/100 Superdex 200 size exclusion column (GE Healthcare, Piscataway, NJ). Protein was eluted at 0.5 ml/min in RJ buffer. The oligomer formation was judged by the appearance of a peak with an earlier retention time. The size exclusion column was calibrated with Thyroglobulin (669 kDa), Ferritin (440 kDa), Aldolase (158 kDa), Conalbumin (75 kDa), Ovalbumin (43 kDa), Carbonic anhydrase (29 kDa), Ribonuclease (137 kDa) and Aprotinin (6.5 kDa). Data analyses were performed using Unicorn 7.0 and were plotted using Origin 7.0.

Microscopy

Confocal imaging was performed using an inverted Olympus FV1000 microscope equipped with the FLUOVIEW 4.2 software. Nucleoprotein images were acquired using a 60 × UPlanSApo/1.35 oil objective with 1-2x magnification. *C. elegans* images were acquired using a UPlanSApo 20x objective with 2x magnification. Imaging settings (laser power and exposure) were set so that negative control images did not show signal. The same microscope settings were then used for all treatments. Sample mounting was not applied prior to imaging.

Super-resolution imaging

Super-resolution SIM (structured illumination microscopy) images were acquired using a Deltavision OMX 3D-SIM System V3 BLAZE from Applied Precision (GE Healthcare) equipped with 3 sCMOS cameras, 405, 488, 592.5 nm diode laser illumination, an Olympus

Plan Apo N 60x 1.42NA oil objective, and standard excitation and emission filter sets. Imaging of each channel was done sequentially using three angles and five phase shifts of the illumination pattern. The refractive index of the immersion oil (Cargille) was adjusted to 1.513 to minimize spherical aberrations. Sections were acquired at 0.125 μm z steps. Raw OMX data were reconstructed in SoftWoRx software version 6.5.2 (Applied Precision, GE Healthcare). Reconstructions were carried out using channel specific Optical Transfer Functions (OTFs) and a Wiener filter of 0.002. OTFs were generated within the SoftWoRx software by imaging 100 nm beads (Life Technologies) using appropriate immersion oils to match the data. For figures, reconstructed data were further processed in FIJI software (open source) to remove negative values created by the reconstruction algorithm. Sample mounting was not applied prior to imaging.

Protein biotinylation

11.8 nmol MRJP-3 and BSA were dialysed in 1xPBS. EZ-Link NHS-PEG$_4$-Biotin (Thermo Scientific; catalog no. 21329) was used to biotinylate the proteins as follows; 170 μl nuclease-free water was added to the EZ-Link NHS-PEG$_4$-Biotin (‘biotin’) powder aliquot and mixed gently. 3 μl of biotin solution was added per 500 μl MRPP-3 or BSA solution, mixed well and incubated at room temperature for 30 min. Biotinylated proteins were then dialyzed in 1xPBS with Slide-A-lyzer MINI dialysis units 7 KDa (Thermo Scientific) for 2 hours followed by buffer change and additional dialysis for 4 hours.

RJ RNA pull down

1 mL raw RJ was diluted with 1.5 mL nuclease-free water and split into two aliquots (1.25 mL per tube). 500 μl of biotinylated protein (MRJP-3 or BSA) was added to each RJ aliquot and rotated overnight at 4 ^{0}c. Next, the RJ pH was adjusted to 5.0-5.5 by adding 0.23 mL of 500 mM Tris pH 8.8. Each RJ-biotinylated protein mixture was then split into two tubes containing 0.4 mL pre-washed Pierce Streptavidin magnetic beads (Thermo Scientific; catalog no. 88816) and rotated at room temperature for 3 hours. Beads were then placed in magnetic stands for 10 min followed by removal of the RJ solutions. Next, beads were washed with 0.5 mL 1xPBS for 10 min at room temperature. After three wash steps, 0.35 mL 1xPBS was added to each beads tube, followed by Phenol/Chloroform/Isoamyl alcohol RNA extraction.

C. elegans soaking

Animals were grown until young adult stage and washed twice in M9 medium. The animals were then transferred into 10 μl treatment solutions placed on a paraffin film, which was sealed within an empty NGM plate and incubated for 2 hours at room temperature. Next, the animals were individually picked and washed three times in M9 medium before fixation in 4% formaldehyde with PBS for 30 min. The animals were then mounted in 1xPBS for imaging.

C. elegans unc-22 RNAi

HT115 empty vector and dsRNA-*unc-22* (ZK617.1) expressing RNAi bacterial feeding clones were kindly received from J. Ahringer’s laboratory, Cambridge University. Bacteria were grown in LB- Ampicillin for 16 hours. The dsRNA-*unc-22* expressing bacteria (positive control for *unc-22* RNAi phenotype) were seeded onto 50 mm NGM agar plates containing 1 mM IPTG and 25 g/ml Carbenicillin at a volume of 50 μl bacterial culture per plate and left to dry for 48 hours. Empty vector bacteria were seeded similarly, but in the absence of IPTG. For *unc-22* dsRNA uptake assays, on day-1, 100 μl of treatment solutions were placed on the center of the empty vector bacterial loan and left to dry for 10 min. Next, 50 L1s synchronized by starvation arrest were spotted onto each plate in a drop of M9. On day-2, second 100 μl of treatment solutions were applied. On day-4, twitcher phenotype was scored at the adult stage. Each treatment had three biological repeats. 5.4 pmol (0.05 μM) dsRNA and 4 nmol (39.7 μM) MRJP-3 or 5 nmol (49.9 μM) MRJP-1 were applied in all dsRNA- and protein-containing treatments in RJ buffer.

Mass spectrometry of royal and worker jelly samples

10% soluble RJ and WJ were dialyzed overnight in 1xPBS buffer, centrifuged at 16 K *rcf.* and passed through a 0.22 μM filter. Jellies proteins were identified by LC-MS/MS applied directly on the RJ and WJ solutions (serviced from Cambridge Centre for Proteomics)

RNA library preparation and sequencing

Total RJ RNA or pulled-down MRJP-3 bound RNA was first subjected to Tobacco Acid Pyrophosphatase (Cap-Clip enzyme, CellScript) and Polynucleotide kinase (T4 PNK, New England Biolabs) treatments according to the manufacturers’ instructions. Total stranded RNA library preparations were performed using the TrueSeq stranded total RNA sample preparation kit (Illumina) according to manufacturer’s instructions omitting the rRNA removal and fragmentation steps since input RNA was of low molecular weight and did not contain obvious rRNA contaminants. In brief, total RJ RNA (ca. 20 ng) or 25% of MRJP-3-bound purified RNA was diluted to 8.5 μl total volume in Elution buffer. After addition of 8.5 μl Elute/Prime/Fragment High Mix (containing the Reverse Transcription primers), RNA was denatured at 65°C for 5 minutes followed by rapid cooling on ice. All subsequent steps were performed following the manufacturer’s protocol with the following modification: PCR amplification of the MRJP-3 bound input cDNA samples was by using 20 cycles in total. All libraries were quantified by standard dsDNA High Sensitivity Qubit assay (Invitrogen) and sizing of libraries was controlled by running 1 μl sample each on a D1000 screen tape using a Tapestation 2200 system (Agilent). Sequencing was performed by a Hiseq 1500 (Illumina, USA) instrument using a 100 bp paired-end read run.

QUANTIFICATION AND STATISTICAL ANALYSIS

RNA-seq analysis

Samples included three total RJ RNA libraries (RJ_1 → RJ-11-9; RJ_2 → RJ-12-22; RJ_3 → RJ-14-3) and two MRJP-3 bound RJ RNA libraries (MRJP-3_1 → MJ3-13-11; MRJP-3_2 → MJ3-8-8). RNA-seq reads were adaptor trimmed using cutadapt v1.11 to a minimum length of 10 nt (Martin, 2011). Trimmed reads were mapped to a combined reference of *Apis mellifera* (GCA_000002195.1) and known honeybee viruses using STAR v2.5.2b (Dobin et al., 2013) with parameters –outFilterMismatchNoverLmax 0.15 –outFilterMultimapNmax 10. Honeybee viruses included Israeli Acute Paralysis Virus (IAPV; accession number NC_009025.1), Acute Bee Paralysis Virus (ABPV; accession number NC_002548.1), Kashmir Bee Virus (KBV; accession number NC_004807.1), Deformed Wing Virus (DWV; accession number NC_004830.2), Varroa Destructor Virus 1 (VDV-1; accession number NC_006494.1), Black Queen Cell Virus (BQCV; accession number NC_003784.1), Sacbrood Virus (SBV; accession number NC_002066.1), Chronic Paralysis Virus (CPV, RNA-1; accession number NC_010711.1), Chronic Paralysis Virus (CPV, RNA-2; accession number NC_010712.1), Bee Macula-like virus (BeeMLV; accession number NC_027631.1), Slow Bee Paralysis Virus (SBPV; accession number NC_014137.1), Lake sinai virus strain-1 (LSV-1; accession number KM886905.1) and Lake sinai virus strain-2 (LSV-2; accession number HQ888865.2). Gene expression was quantified using featureCounts v1.5.0-p2 from Subread (Liao et al., 2013) on gene level. For plotting, counts were summed over each species and, in bees, RNA biotype. To compare counts across biological samples, library size factors were calculated as the fraction of each sample's total mapped read count divided by the mean sample read count. Afterward, each sample's counts were normalized by dividing by the sample library size factor. Finally, we tested whether read counts between *A. mellifera* and *Varroa destructor virus-1* were significantly different, both for the total royal jelly fraction and the MRJP-3 fraction, using a two-sided Welch two-sample t test.

Viral coverage

RNA-seq coverage of the *Varroa Destructor Virus-1* was computed strand-specifically using bedtools genomecov 2.27.1 (Quinlan and Hall, 2010). Coverage was plotted on a log scale by performing the transformation $\log_{10}(x+1)$ on counts x (and flipping the axis for the antisense strand).

GO analysis

For GO analysis, genes were considered as expressed if they had a read count after RNA-seq mapping and quantification of at least 2. We considered only GO terms with at least 5 annotated genes; GO terms were downloaded from Ensembl Metazoa Biomart. A null distribution was calculated from expressed genes using the gene lengths (as the mean length of a gene's transcripts) as a bias term (via goseq 1.28 (Young et al., 2010)). GO terms were called overrepresented when their Wallenius hypergeometric test p value was < 0.05.

MicroRNA screen

To discover unannotated microRNAs, reads that mapped to the *A. mellifera* genome were re-mapped against the "hairpin.fa" pre-miRNA reference from miRBase, release 21 as single-end reads, using STAR with parameters –outFilterMismatchNoverLmax 0.15 –outFilterMismatchNmax 1 –alignIntronMax 1 –scoreDelOpen -10000 –scoreInsOpen -10000 –outFilterMultimapNmax 100.

Double-stranded honeybee RNA screen

Reads that mapped to the *Apis mellifera* genome were analyzed for the occurrence of putative honeybee dsRNA. Long RNA-seq data were split into first and last read of each fragment. Subsequently, we tested for pairwise overlaps of reads on the forward and reverse strand via bedtools intersect 2.27.1 (Quinlan and Hall, 2010), where the overlap was at least 25 nt, and the overhang on either side did not exceed 100 nt. We quantified the count of dsRNA candidates falling on each annotated gene (normalized by their library size as described in DESeq (Anders and Huber, 2010)). We further classified unique dsRNA candidates (characterized here by unique start and end coordinates) by noting their length distribution as well as classifying the gene annotation of their loci.

DATA AND SOFTWARE AVAILABILITY

The RNA-seq data have been deposited in the ArrayExpress database at EMBL-EBI under accession number E-MTAB-6732. All in-house scripts have been deposited in Github and can be downloaded: https://github.com/klmr/royal-jelly (https://doi.org/10.5281/zenodo.1542860). Other Software used in this work are all publicly available, with the links to them in the above tables. The raw imaging data, including images of gels and blots, have been deposited in Mendeley Data and can be accessed: https://doi.org/10.17632/5w7rbd8452.1. All the rest of the data are available in the manuscript or the supplementary materials.

Molecular Cell, Volume *74*

Supplemental Information

A Secreted RNA Binding Protein Forms RNA-Stabilizing Granules in the Honeybee Royal Jelly

Eyal Maori, Isabela Cunha Navarro, Humberto Boncristiani, David J. Seilly, Konrad Ludwig Moritz Rudolph, Alexandra Sapetschnig, Chi-Chuan Lin, John Edward Ladbury, Jay Daniel Evans, Jonathan Luke Heeney, and Eric Alexander Miska

SUPPLEMENTAL INFORMATION

Fig. S1 (related to Fig. 1) | Royal jelly proteins bind polymeric nucleic acids

(A) Assessment of Alexa Fluor-488 labeled dsRNA (dsRNA*) stability in hive conditions to control for contaminating leakage of dsRNA solution into the newly secreted RJ. 50%, 25% and 10% sucrose solutions (w/v) were exposed to living bees for 30 min. Next, the sucrose solutions were mixed with dsRNA* (50 ng/ul final concentration) and incubated within hives for 0, 24, 48, 72 and 96 hours. dsRNA* integrity was analysed by gel electrophoresis of 10 μl samples. dsRNA* mixed in 10% sucrose solution could not be detected after 96 hours. Thus, dsRNA* was applied in 10% sucrose solution, and RJ was harvested 96 hours after the last dsRNA* application. **(B)** RT-PCR detection of dsRNA* in RJ samples harvested from control and dsRNA*-fed hives. **(C)** Worker jelly (WJ) proteins bind dsRNA. DsRNA-binding activity was tested by Electrophoretic Mobility Shift Assay (EMSA). Treatments included dsRNA mixed in RJ-buffer, 10% WJ mixed with dsRNA, 10% WJ was digested by PK and then mixed with dsRNA, 10% WJ was mixed with dsRNA and then digested by PK, 10% WJ was mixed with dsRNA and PK buffer, purified 27.3 μM BSA mixed with dsRNA, 10% WJ only, 10% WJ only digested by PK. 0.05 μM dsRNA was applied in all dsRNA-containing treatments. **(D)** The RJ dsRNA-binding proteins are soluble. dsRNA-binding activity was tested by EMSA. Treatments included dsRNA mixed in RJ buffer, 10% raw RJ mixed with dsRNA, 10% soluble RJ fraction mixed with dsRNA. 0.05 μM dsRNA was applied in all treatments. **(E)** Free nucleotides do not interfere with dsRNA-binding activity of RJ proteins. DsRNA-binding was tested in the presence of increasing concentrations of the negatively charged deoxynucleotides or nicotinamide adenine dinucleotide (NAD). 0.05 μM dsRNA was applied in all treatments. **(F)** The effect of RJ dilution factor on dsRNA band shift profile. DsRNA binding-activity was tested by EMSA. Constant dsRNA concentration was introduced to different RJ concentrations. Treatments included dsRNA mixed in RJ-buffer only and different raw and soluble RJ dilutions mixed with dsRNA. 0.05 μM dsRNA was applied in all treatments. **(G)** RJ proteins:dsRNA ratio affects dsRNA band shift profile. dsRNA-binding activity was tested by EMSA. Constant RJ concentration was introduced to increasing concentrations of dsRNA. Treatments included dsRNA mixed in RJ-buffer only and different

dsRNA concentrations mixed with 2% raw RJ (upper gel). In the gel below, gel electrophoresis of soluble RJ that was extracted from each sample post dsRNA mixture.

Fig. S2 (related to Fig. 2) | MRJP-3 forms an oligomeric structure

(A) MRJP-3 binds similarly 50 nt ssRNA and dsRNA carrying the same sequence. 42.8 μM proteins and 0.27 μM ssRNA or dsRNA were used in all RNA- and/or protein-containing treatments. **(B)** Purified MRJP-1 does not bind dsRNA as demonstrated by EMSA. dsRNA was incubated with decreasing concentrations of MRJP-3 and MRJP-1. Additional controls: MRJP-3 only, MRJP-1 only and dsRNA only. 0.08 μM dsRNA was applied in all dsRNA-containing treatments. **(C)** Graphical representation of MRJP-1 and MRJP-3. **(D)** Gel filtration analysis determines MRJP-3 molecular weight in RJ buffer. **(E)** Binding curve of Alexa Fluor-488 labeled MRJP-3 to MRJP-3 in RJ buffer. Calculated equilibrium disassociation constant (Kd) value is shown in dashed lines. **(F)** Evaluation of MRJP-3's concentration in RJ by comparative band intensity. RJ-1 and RJ-2: Two RJ samples collected from different hives. Equal sample volumes were loaded in all wells. Red and green spots represent treatments with similar band intensity. **(G)** The tandem-repeats region of MRJP-3 is required for RNA-binding. ssRNA-binding activity was tested by EMSA. Treatments included ssRNA only, full-length recombinant MRJP-3 (rMRJP-3) mixed with ssRNA, recombinant MRJP-3 lacking the repeats region (rMRJP3Δrepeats) mixed with ssRNA, rMRJP-3 mixed with ssRNA followed by proteinase K (PK) digestion, rMRJP-3 only. 0.3 μM ssRNA and 13.65 μM proteins were applied in all ssRNA- and/or protein-containing treatments.

Fig. S3 (related to Fig. 3) | RNA mediates super-order assembly of MRJP-3 oligomers into large RNPs, and isolation of royal jelly RNA partners of MRJP-3

(A) Super resolution OMX imaging of RNPs formed by MRJP-3 interaction with Alexa Fluor-488 labeled ssRNA or dsRNA. 0.2 μM ssRNA* or 0.03 μM dsRNA* were introduced to 15 μM unlabeled MRJP-3. Scale bar represents 1 μm. **(B)** RNA mediates super-order assembly of MRJP-3 oligomers, resulting in large RNPs formation. 0.15 μM ssRNA or ssRNA* were introduced to 42.8 μM unlabeled or Alexa Fluor-633 labeled MRJP-3 (MRJP-3 or MRJP-3* respectively). Scale bar represents 2 μm. **(C)** Bioanalyzer

Electropherograms of MRJP-3 bound RJ RNA. Complexes of RNA and biotinylated MRJP-3 were pulled down with strepatividine coated magnetic beads. Treatments also included RNA pull-down with biotinylated BSA or with beads only. **(D)** VDV-1 coverage plot. The y-axis shows the per-base read coverage for each RNA-seq library across the genome of VDV-1 (green: MRJP-3 bound RNA; blue: total RJ RNA). Positive and negative coverage values represent viral RNA that corresponds to the plus (sense) or minus (antisense) VDV-1 genome, respectively. **(E)** MRJP-3 binds putative long tRNA-dsRNA fragments (≥25 bp).

Fig. S4 (related to Fig. 4) | MRJP-3 bound dsRNA is protected from digestion of RNaseA, but not RNaseIII

(A) MRJP-3 bound dsRNA is protected from RNaseA digestion. Treatments included dsRNA mixed with MRJP-3, dsRNA mixed with MRJP-3 followed by incubation with RNaseA, dsRNA mixed with MRJP-1, dsRNA mixed with MRJP-1 followed by incubation with RNaseA, dsRNA mixed in RJ buffer and dsRNA mixed in RJ buffer followed by incubation with RNaseA. 0.04 µM dsRNA and 42.8 µM MRJP-3 or MRJP-1 were used in all dsRNA- and protein-containing treatments. RNase challenge was performed by introducing 0.2 ug RNaseA followed by 3 hours incubation at room temperature. **(B)** MRJP-3 bound dsRNA is digested by RNase-III. Treatments included dsRNA mixed with MRJP-3, dsRNA mixed with MRJP-3 followed by incubation with RNase-III, dsRNA mixed with MRJP-1, dsRNA mixed with MRJP-1 followed by incubation with RNase-III, dsRNA mixed in RJ buffer and dsRNA mixed in RJ buffer followed by incubation with RNase-III. 0.04 µM dsRNA and 42.8 µM MRJP-3 or MRJP-1 were used in all dsRNA- and protein-containing treatments. RNase-III challenge was performed by introducing $2x10^{-2}$ units RNase-III followed by 3 hours incubation at room temperature. **(C)** MRJP-3 RNPs are susceptible to RNase-III. Three images of RNPs formed with dsRNA* with or without RNase-III. 0.04 µM dsRNA* and 42.8 µM MRJP-3 were used in all dsRNA*- and protein-containing treatments. RNase challenge was performed by introducing $2x10^{-2}$ units RNase-III followed by 3 hours incubation in room temperature. Scale bar represents 20 µm. **(D)** Labelled dsRNA stability in animals soaking assay conditions. Gel electrophoresis of animals soaking solution treated with PK.

Fig. S1

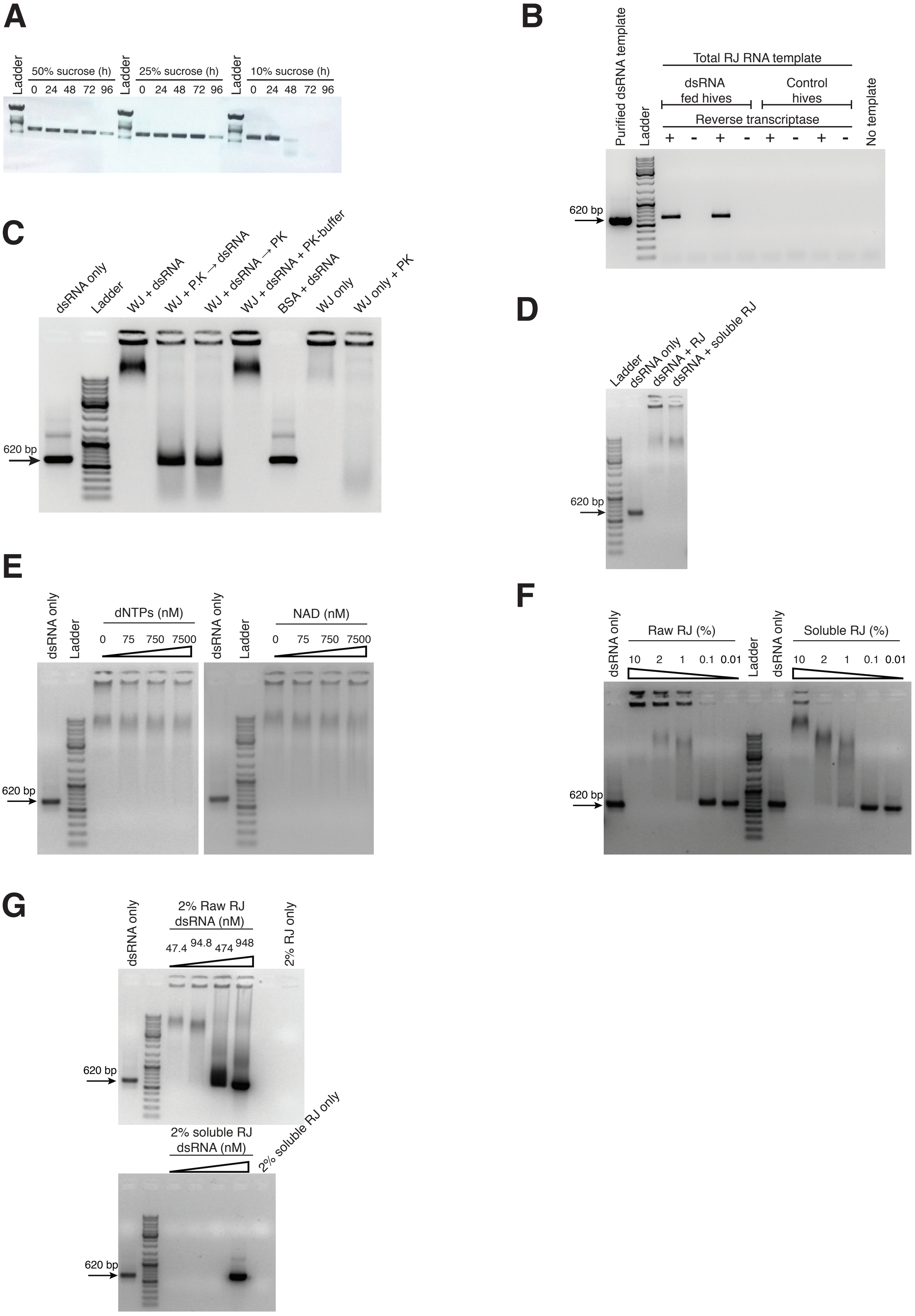
A
Ladder
50% sucrose (h)
0 24 48 72 96
25% sucrose (h)
10% sucrose (h)
B
Purified dsRNA template
Total RJ RNA template
dsRNA fed hives
Control hives
Reverse transcriptase
No template
620 bp
C
dsRNA only
WJ + dsRNA
WJ + P.K → dsRNA
WJ + dsRNA → PK
WJ + dsRNA + PK-buffer
BSA + dsRNA
WJ only
WJ only + PK
D
dsRNA + RJ
dsRNA + soluble RJ
E
dNTPs (nM)
0 75 750 7500
NAD (nM)
F
Raw RJ (%)
10 2 1 0.1 0.01
Soluble RJ (%)
G
2% Raw RJ
dsRNA (nM)
47.4 94.8 474 948
2% RJ only
2% soluble RJ
2% soluble RJ only

Fig. S2

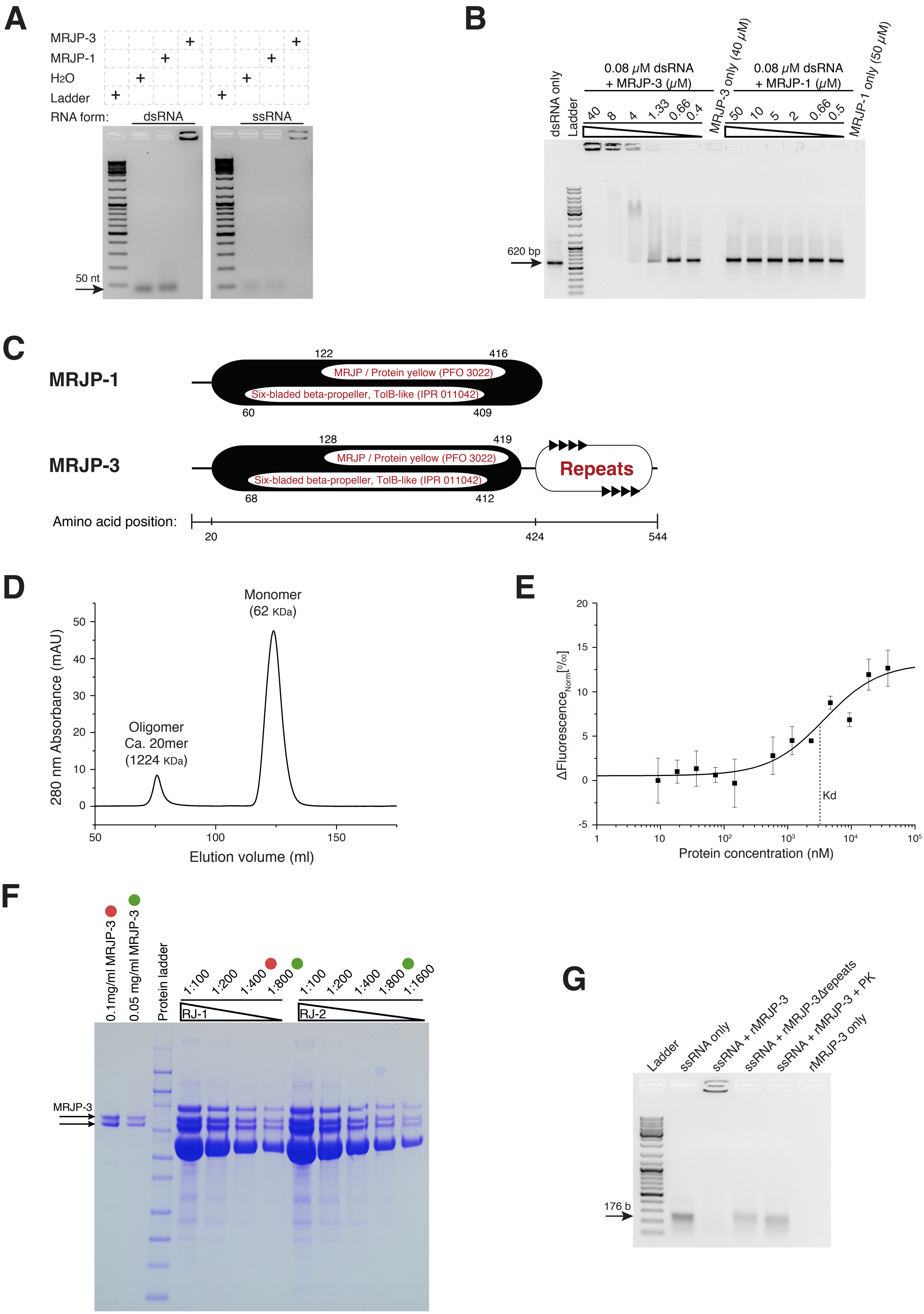

A
MRJP-3
MRJP-1
H2O
Ladder
RNA form:
dsRNA
ssRNA
50 nt
B
dsRNA only
Ladder
0.08 μM dsRNA + MRJP-3 (μM)
40 8 4 1.33 0.66 0.4
MRJP-3 only (40 μM)
0.08 μM dsRNA + MRJP-1 (μM)
50 10 5 2 0.66 0.5
MRJP-1 only (50 μM)
620 bp
C
MRJP-1
122 416
MRJP / Protein yellow (PFO 3022)
Six-bladed beta-propeller, TolB-like (IPR 011042)
60 409
MRJP-3
128 419
MRJP / Protein yellow (PFO 3022)
Six-bladed beta-propeller, TolB-like (IPR 011042)
68 412
Repeats
Amino acid position:
20 424 544
D
Monomer (62 KDa)
Oligomer Ca. 20mer (1224 KDa)
280 nm Absorbance (mAU)
Elution volume (ml)
E
ΔFluorescence Norm [‰]
Kd
Protein concentration (nM)
F
0.1mg/ml MRJP-3
0.05 mg/ml MRJP-3
Protein ladder
1:100 1:200 1:400 1:800
RJ-1
1:100 1:200 1:400 1:800 1:1600
RJ-2
MRJP-3
G
Ladder
ssRNA only
ssRNA + rMRJP-3
ssRNA + rMRJP-3Δrepeats
ssRNA + rMRJP-3 + PK
rMRJP-3 only
176 b

Fig. S3

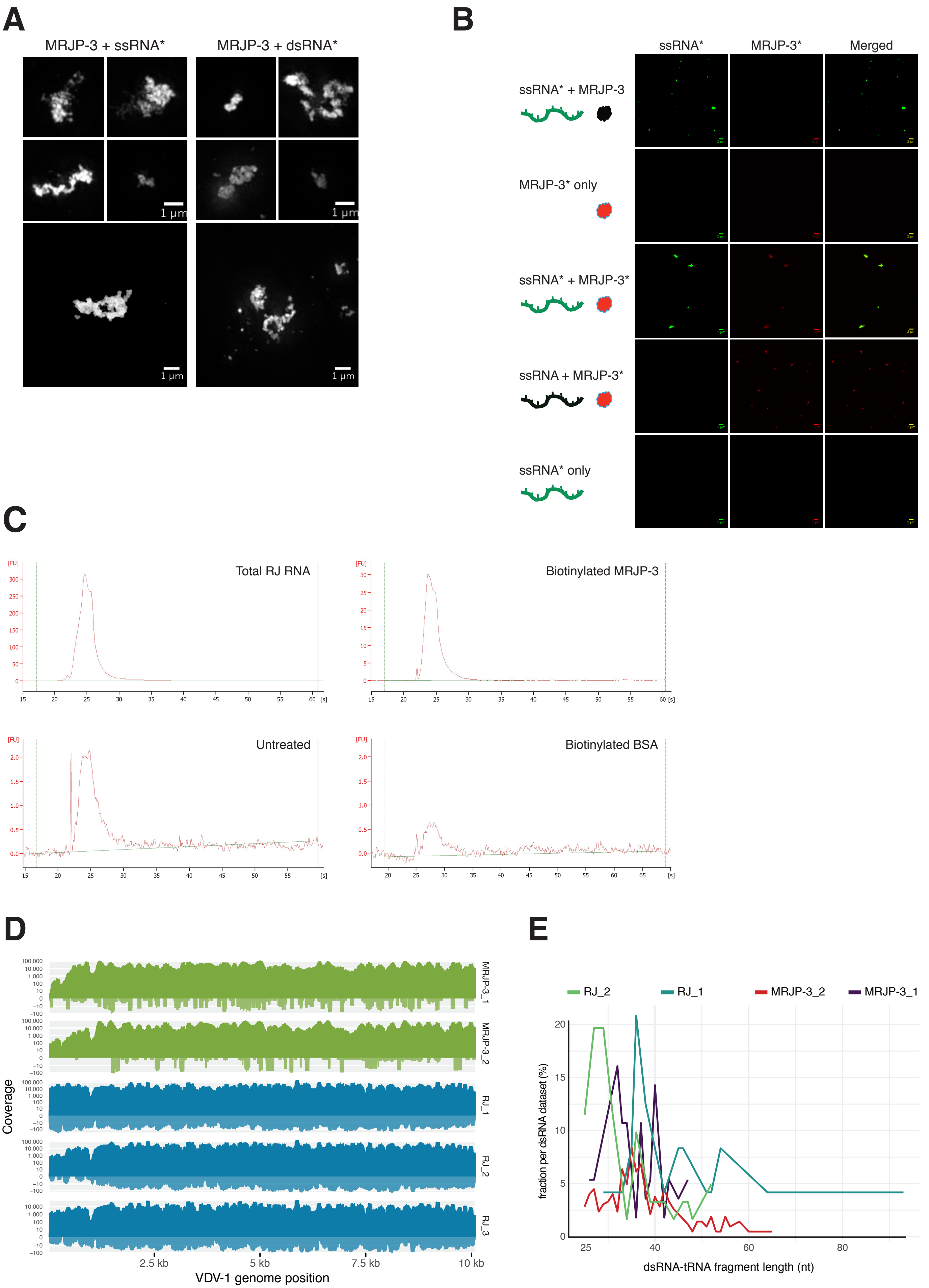

A
MRJP-3 + ssRNA*
MRJP-3 + dsRNA*
1 µm
B
ssRNA*
MRJP-3*
Merged
ssRNA* + MRJP-3
MRJP-3* only
ssRNA* + MRJP-3*
ssRNA + MRJP-3*
ssRNA* only
C
Total RJ RNA
Biotinylated MRJP-3
Untreated
Biotinylated BSA
[FU]
[s]
D
Coverage
MRJP-3_1
MRJP-3_2
RJ_1
RJ_2
RJ_3
2.5 kb
5 kb
7.5 kb
10 kb
VDV-1 genome position
E
RJ_2
RJ_1
MRJP-3_2
MRJP-3_1
fraction per dsRNA dataset (%)
dsRNA-tRNA fragment length (nt)

Fig. S4

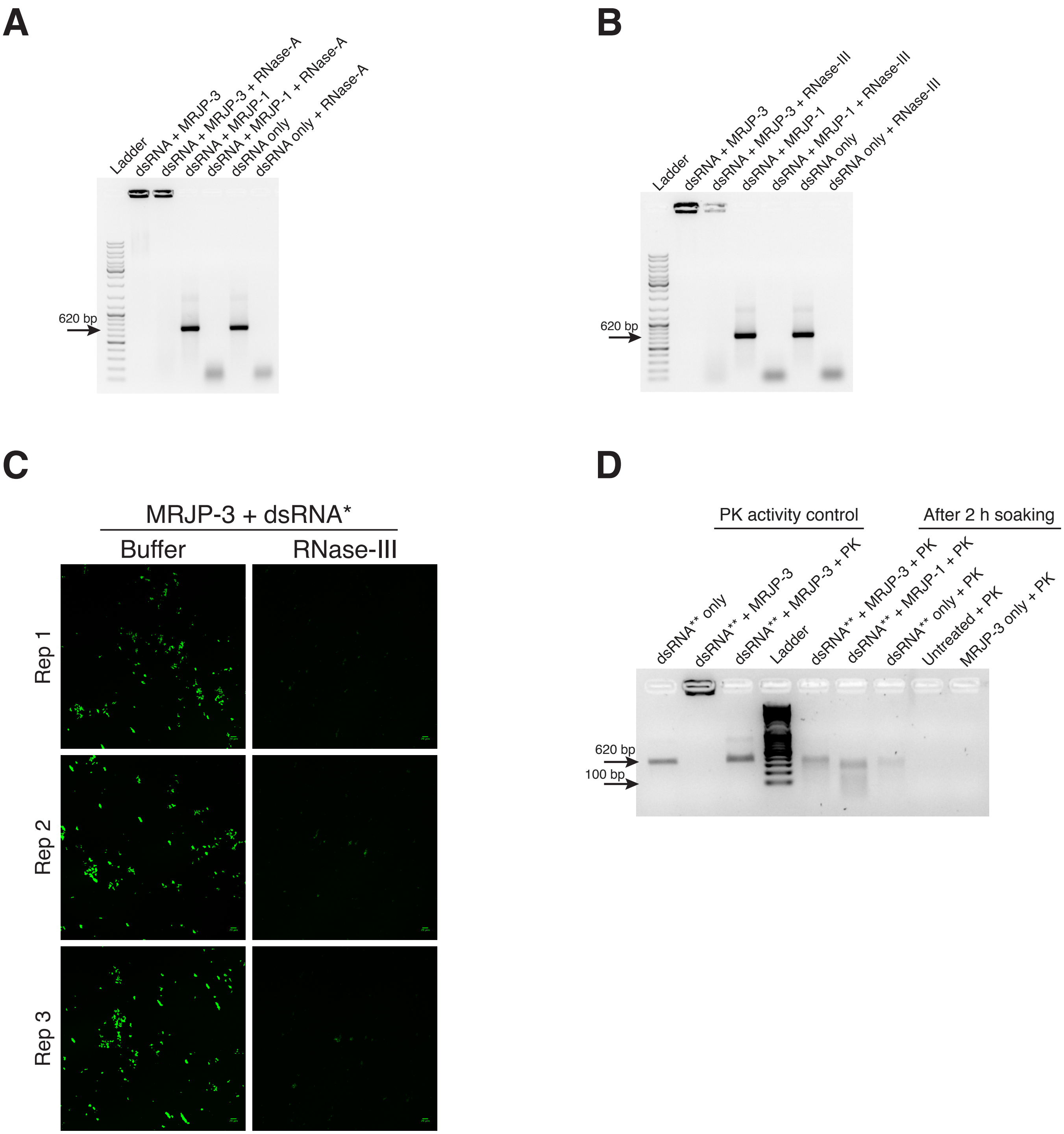
A
Ladder
dsRNA + MRJP-3
dsRNA + MRJP-3 + RNase-A
dsRNA + MRJP-1
dsRNA + MRJP-1 + RNase-A
dsRNA only
dsRNA only + RNase-A
620 bp
B
Ladder
dsRNA + MRJP-3
dsRNA + MRJP-3 + RNase-III
dsRNA + MRJP-1
dsRNA + MRJP-1 + RNase-III
dsRNA only
dsRNA only + RNase-III
620 bp
C
MRJP-3 + dsRNA*
Buffer
RNase-III
Rep 1
Rep 2
Rep 3
D
PK activity control
After 2 h soaking
dsRNA** only
dsRNA** + MRJP-3
dsRNA** + MRJP-3 + PK
Ladder
dsRNA** + MRJP-3 + PK
dsRNA** + MRJP-1 + PK
dsRNA** only + PK
Untreated + PK
MRJP-3 only + PK
620 bp
100 bp
